# Multiple metals in children’s deciduous teeth: results from a community-initiated pilot study

**DOI:** 10.1038/s41370-021-00400-x

**Published:** 2021-11-08

**Authors:** Alexa Friedman, Julia Anglen Bauer, Christine Austin, Timothy J. Downs, Yorghos Tripodis, Wendy Heiger-Bernays, Roberta F. White, Manish Arora, Birgit Claus Henn

**Affiliations:** 1 Department of Environmental Health, Boston University School of Public Health, Boston, MA USA; 2 Department of Epidemiology, Geisel School of Medicine, Dartmouth College, Lebanon, NH USA; 3 Department of Environmental Medicine and Public Health, Icahn School of Medicine at Mount Sinai, New York, NY USA; 4 Department of International Development, Community, and Environment, Clark University, Worcester, MA USA; 5 Department of Biostatistics, Boston University School of Public Health, Boston, MA USA; 6 Department of Neurology, Boston University School of Medicine, Boston, MA USA

**Keywords:** Metals, Early-life exposure, Biomonitoring, Child exposure/health

## Abstract

**Background:**

Characterizing retrospective exposure to toxicants during multiple early-life developmental periods is challenging, yet critical for understanding developmental effects.

**Objective:**

To characterize early-life metal exposure using deciduous teeth in a community concerned about past exposures.

**Methods:**

Naturally shed teeth were collected from 30 children ages 5–13 years who resided in Holliston, Massachusetts since conception. We estimated weekly prenatal and postnatal (up to 1 year of age) exposure to 12 metals by measuring dentine concentrations using laser ablation-inductively coupled plasma-mass spectrometry. Multivariable linear mixed models were used to explore sociodemographic, dietary, and behavioral correlates of dentine metal concentrations.

**Results:**

Temporal trends in dentine levels differed by metal. Source of milk during the first year of life was associated with dentine barium (Ba) levels, where being fed predominantly breastmilk was associated with 39% (95% CI: –57%, –13%) lower dentine Ba compared to predominantly formula use. Females had higher prenatal and postnatal dentine Mn and Pb, compared to males (e.g., % difference, postnatal Mn: 122% (17%, 321%); postnatal Pb: 60% (95% CI: –8%, 178%)).

**Significance:**

Deciduous teeth provide retrospective information on dose and timing of early-life metals exposure at high resolution. We demonstrate their utility in a community-based study with known past contamination of drinking water.

**Impact statement:**

We conducted a community-initiated pilot study in a community concerned with historical exposure to multiple metals. Using deciduous teeth, a novel noninvasive biomarker, we characterized early-life exposure to 12 metals in approximately weekly increments during sensitive developmental periods, thus demonstrating the utility of this biomarker in communities concerned with past exposures.

## Introduction

Critical windows of susceptibility, also referred to as critical windows of exposure, are periods during development in which exposures to specific toxicants have a greater effect on a health outcome compared to the same exposure during other periods [1]. During development, the brain undergoes unique changes in morphology and connectivity, making gestation and infancy critical periods during which exposure to toxicants often have adverse effects on neurodevelopment [2, 3]. Furthermore, protective regulatory mechanisms such as the blood–brain barrier are not fully formed, making the fetus and infant particularly susceptible to overexposure to toxicants [4, 5]. Increasing evidence points to heterogeneity in the health effects of metals depending on timing of exposure [5, 6]. Thus, it is important to characterize metal exposure during multiple developmental periods, rather than at a single time point.

Measuring retrospective exposure, particularly during critical periods such as in utero and early childhood, presents a challenge in environmental epidemiology studies. While estimates of past exposure, such as historic public drinking water or air monitoring data, are sometimes available that coincide with the critical windows of interest, these environmental measures have greater error compared to internal biomarkers for measuring individual-level exposure, given that they are further removed from the target tissue dose [7]. Traditional biomarkers of prenatal and early-life exposure (e.g., maternal or cord blood) are typically collected less frequently, are limited in the information they provide (i.e., may represent shorter exposure windows), and are subject to exposure misclassification (i.e., do not reflect direct exposure to the fetus). Collection of other biospecimens (e.g., hair, nails, urine) from infants and young children can be logistically difficult and would be required at multiple time points after birth, as these biospecimens represent relatively short windows of exposure.

The use of deciduous, or naturally shed, baby teeth presents an opportunity to assess retrospective early-life exposure to toxicants in a highly temporally resolved manner and thus allows for estimation of exposure in a noninvasive sample during relevant and sensitive developmental windows [8, 9]. Metals accumulate in tooth dentine beginning in the second trimester of pregnancy and tooth mineralization occurs over time, spanning the prenatal and early childhood periods [8, 9]. At birth, the neonatal line, a histological feature, is formed in the tooth dentine, allowing researchers to characterize metal exposure during the prenatal, postnatal, and early childhood periods with temporal precision [8, 9]. Furthermore, a single tooth provides high-resolution repeated measures of exposure that increases the amount of data per individual and avoids the logistical challenges of collecting repeated biospecimens, which is necessary when examining critical windows of susceptibility using other biomarkers.

Retrospective biomarkers that can measure levels of multiple contaminants are particularly useful in settings where it is necessary to quantify internal levels of numerous known prior exposures. For example, regulated (e.g., Pb, having an Action Level under the Safe Drinking Water Act) and unregulated (e.g., Mn, with only a non-enforceable guideline in drinking water) metals have been detected in public drinking water supplies in the US and globally at levels that may pose a public health risk [10–12]. Increasing evidence links exposure to metals in drinking water with decrements in neurobehavioral outcomes in children [11] but the levels of unregulated contaminants, like Mn, in drinking water that are considered safe, particularly for vulnerable subpopulations like children, are unknown [11, 13]. Some limitations of previous epidemiological studies of metals in drinking water include the lack of a consistent biomarker across studies and the inability to measure past exposure during the most relevant exposure windows [11]. Thus, the application of the tooth biomarker may be particularly useful in the context of known past contamination and in community-based studies, in which sample sizes are often small, there may be multiple contaminants of concern, and it is rarely possible to reconstruct personal exposures accurately [14].

We conducted a community-initiated pilot study in the suburban town of Holliston, Massachusetts (MA), USA. In Holliston, the community relies on two local aquifers for nearly 100% of their drinking water, which are susceptible to landfill and industrial contamination and contain naturally elevated levels of Mn and other metals, as evidenced by episodic discolored residential tap water that has occurred for years [15]. This study was conducted in response to residents’ concerns about early-life exposure to metals and their children’s health. The study aims to assess children’s exposure during critical periods of development, with regular bidirectional communication with community members. By estimating exposure to multiple metals during sensitive life stages (in utero through 1 year of life) using deciduous teeth as a novel, high-resolution biomarker of retrospective exposure, we conducted an exploratory analysis to examine demographic, behavioral, and water consumption factors as correlates of dentine metal concentrations.

## Materials and methods

### Study area and recruitment

Participants were part of the Assessing Children’s Environmental Exposures (ACHIEVE) study, a community-initiated pilot research study in Holliston, MA, USA. Mother–child pairs were recruited through local newspapers, posting of brochures on town bulletins, community Facebook pages, and word of mouth. Eligibility criteria included: being a resident of Holliston, MA, at time of enrollment; having lived in Holliston during pregnancy with participating child; having a child between 5 and 13 years old at time of enrollment who had lost or were losing teeth; and being willing to donate their child’s shed tooth. If multiple children in a household were eligible, one child per household was included in the study, selected at random.

Eligible participants were informed about the study and written consent was obtained from mothers prior to participation. Of 35 mother–child pairs who expressed interest in the study, 5 were ineligible because the child was either outside of the eligible age range (*n* = 2) or did not have a deciduous tooth to donate (*n* = 3). A total of 30 mother–child pairs were enrolled between 2017 and 2018, 29 of whom donated a tooth prior to analysis in 2018. Home visits were conducted to collect deciduous teeth and administer questionnaires to mothers. Information was obtained via questionnaire on participant sociodemographic characteristics, drinking water consumption patterns, use of filters for drinking water, sources of milk during infancy, and parent-reported learning and behavioral disorders. The research study protocol and all study materials were approved by the Institutional Review Board at the Boston University School of Public Health.

### Metals in deciduous teeth

One deciduous tooth was collected per child during the home visit. Molars and incisors that had been shed prior to or during enrollment were collected and stored at room temperature before being shipped to the Senator Frank R. Lautenberg Health Sciences Laboratory at the Icahn School of Medicine at Mount Sinai for analysis of metals content. Teeth that had defects or high levels of attrition (i.e., excessive wear and tooth surface loss) (*n* = 1) were unable to be analyzed and were thus excluded. Analytic methods for measurement of dentine metal concentrations have been validated and described previously in detail [8, 16, 17]. Using the neonatal line formed at birth, incremental markings on the teeth akin to growth rings in trees are used to assign temporal sampling points (about every 7–10 days). Using laser ablation-inductively coupled plasma-mass spectrometry (LA-ICP-MS), signal intensities (i.e., values) of the following 12 metals were measured: Mn, Pb, barium (Ba), chromium (Cr), cobalt (Co), copper (Cu), lithium (Li), magnesium (Mg), molybdenum (Mo), strontium (Sr), tin (Sn), and zinc (Zn). The panel of metals represents the standard panel that can be reliably measured by LA-ICP-MS in the tooth matrix. Calcium (Ca) was also measured to account for individual mineral density variation within and between participants and metal values were normalized to Ca ion values (e.g., ^55^Mn:^43^Ca ratio for manganese). The metal value in the gas blank was subtracted from the metal values measured in each tooth prior to normalizing to Ca. Tooth metal values that are less than levels measured in the blank (and thus are negative values) represent levels below the limit of detection (LOD). Detection frequency was above 97% for all metals except Co and Mo (78% and 89%, respectively) (Supplementary Table 1). Given the relatively low percentage of values below the LOD [18], samples below the LOD were set to the metal’s lowest measured level divided by square root of 2 (Supplementary Table 1). To allow for a comparison of dentine metal levels between this study and other populations, an external standard correction was used to account for differences between laboratories and analytical methods. The certified reference material NIST 610 was applied to the dentine metals data for external correction. Laboratory technicians were blinded to participant information.

### Potential correlates of dentine metal concentrations

The following demographic, dietary, and behavioral characteristics were considered as potential correlates of dentine metal concentrations: child sex, birth order, age (continuous, in years), public versus private drinking water source, filter use during pregnancy, tooth type (incisors versus molars), maternal water consumption habits during pregnancy, maternal anemia during pregnancy, infant formula use, and breastfeeding. For water consumption habits during pregnancy, mothers reported their frequency of use of filtered water for food preparation, filtered water for making coffee/tea, and bottled water. Mothers reported duration of breastfeeding (<3 months, 3–6 months, 6–9 months, 9 months–1 year, >1 year), ever use of formula in infant’s first year, infant’s age at first use of formula, and percentage of milk in infant’s first year that was formula (<10%, 10–<50%, 50%, >50–<100%, 100%). Information on breastfeeding duration and percentage of milk that was formula in infant’s first year were combined to estimate the predominant source of milk in infant’s first year (breastmilk, formula, or mix). Given the small sample size for some of these groups, the following correlates are presented in supplementary tables only: well type, filtered water use for food preparation, filtered water use for coffee/tea preparation, bottled water use, and breastfeeding (ever). For temporality reasons, source of milk was evaluated as a correlate of postnatal levels of dentine metals only.

### Statistical analyses

Summary statistics were calculated, and distributions were examined for all variables (Tables 1 and 2). Dentine metal levels, measured as a ratio of metal to calcium (i.e., metal:Ca), represent approximately weekly measurements of exposure for each participant from the second trimester of pregnancy through 1 year of age. For summary statistics only, we averaged the fine-resolution tooth metal measurements within each subject for each of two exposure periods: prenatal (second trimester to birth) and postnatal (birth through 1 year of age) (Supplementary Table 2).

**Table 1:** Sociodemographic characteristics of participating mother–child pairs (*n* = 28).

Child	*N* (%) or mean (range)
*Sex*	
Male	18 (64)
Female	10 (36)
*Age, years*	8.8 (5.8–14.5)
*Has siblings*	26 (93)
*Birth order*	
First	13 (46)
Second or higher	15 (54)
*Requires special services at school*	9 (32)
*Has an individualized education program*	8 (29)
*Learning disability*^a^	6 (21)
*Attention deficit hyperactive disorder*^a^	1 (4)
*Autism spectrum disorder*^a^	4 (14)
*Family has moved since pregnancy (within Holliston)*	5 (18)
*Breastfed, ever*	25 (89)
*Duration of breastfeeding*	
<3 months	5 (20)
3–6 months	4 (16)
6–9 months	3 (12)
9 months–1 year	3 (12)
>1 year	10 (40)
*Formula use, ever*	21 (75)
*Percent of milk that was formula in first year of life*	
<10	1 (5)
10–<50	4 (19)
50	4 (19)
>50–100	4 (19)
~100	8 (38)
*Source of water for formula preparation*	
Tap water (unfiltered)	8 (38)
Tap water (filtered)	12 (57)
Bottled water	0 (0)
Missing	1 (5)
*Predominant source of milk in first year of life*	
Formula^b^	11 (39)
Breastmilk^c^	11 (39)
Mix of breastmilk and formula^d^	6 (21)
*Tooth type*	
Incisor	24 (86)
Molar	4 (14)

Mother	*N* (%)
*Race/ethnicity*	
Non-Hispanic white	26 (93)
Other, multiracial	2 (7)
*Employed outside home during pregnancy*	25 (89)
* Worked in Holliston during pregnancy*	1 (4)
Anemic during pregnancy^e^	
Yes	8 (29)
No	19 (68)
Don’t know	1 (4)

^a^Parent-reported physician diagnosis.

^b^Predominantly formula defined as maternal report of being breastfed less than 6 months and consuming ≥50% formula during first year of life.

^c^Predominantly breastmilk defined as maternal report of being breastfed longer than 6 months and consuming less than 50% formula during first year of life.

^d^Mix of breastmilk and formula defined as maternal report of being breastfed for less than 6 months and consuming less than 50% formula during first year of life OR maternal report of being breastfed for longer than 6 months and consuming ≥50% formula during first year of life.

^e^Ever told by physician during pregnancy.

**Table 2:** Drinking water characteristics and consumption patterns during pregnancy (*n* = 28).

	*N* (%)
*Household water source*	
Public water supply	25 (89)
Private well	3 (11)
*Has filtration system*	
Yes	14 (50)
Point of entry (basement)	2 (14)
Point of use (sink, pitcher)	12 (86)
No	14 (50)
*Use of filtered water for food preparation*	
Always	3 (11)
Rarely/sometimes	4 (14)
Never	21 (75)
*Use of filtered water for coffee/tea preparation*	
Always	6 (21)
Rarely/sometimes	6 (21)
Never	16 (59)
*Bottled water use*	
Always	2 (7)
Rarely/sometimes	17 (61)
Never	9 (32)

To investigate correlates of dentine metal levels, we used a multivariable linear mixed model with the fine-resolution dentine metal values (approximately weekly measurements) as the dependent variable. We examined correlates separately for each developmental period, given the potential for distinct associations in these two different windows. Thus, two sets of models were fit: one for the prenatal period and one for the postnatal period (i.e., weekly measurements from the second trimester until birth as the dependent variable in prenatal models; from birth to 1 year of age in postnatal models). Dentine metal values were right skewed; we log-transformed metal values to reduce the influence of extreme values and to meet the normality of residuals assumption for linear regression. A subject-specific random intercept accounted for within-subject correlation of repeated weekly measures of dentine metal values. All models were adjusted for child sex. Percent difference (%D) in dentine metal value per unit increase in the correlate was calculated by exponentiating the beta coefficient, subtracting 1, and then multiplying by 100 (i.e., (e^beta^ − 1) × 100)) (Tables 3–5 and Supplementary Tables 3–14). In a sensitivity analysis, we computed associations between correlates and tooth levels truncated to postnatal week 20, given the reduced number of sampling points that could be collected from teeth in our sample after 20 weeks post birth (Supplementary Table 14).

**Table 3:** Correlates of log-transformed prenatal and postnatal dentine Ba levels (as ^138^Ba:^43^Ca) from sex-adjusted linear mixed models.

		Prenatal Ba		Postnatal Ba	
	*N*	*β* (95% CI)	% Difference (95% CI)	*β* (95% CI)	% Difference (95% CI)
*Child sex*					
Male	18	ref	ref	ref	ref
Female	10	0.1 (–0.2, 0.5)	15 (–14, 55)	0.1 (–0.2, 0.5)	13 (–21, 60)
*Child age, continuous (years)*	28	0.02 (–0.1, 0.1)	2 (–5, 8)	0.01 (–0.1, 0.1)	2 (–6, 10)
*Birth order*					
Second or higher	15	ref	ref	ref	ref
First	13	0.1 (–0.3, 0.4)	7 (–22, 47)	–0.01 (–0.4, 0.4)	–1 (–32, 45)
*Tooth type*					
Incisor	24	ref	ref	ref	ref
Molar	4	0.1 (–0.3, 0.5)	10 (–27, 66)	0.5 (–0.5, 0.6)	5 (–36, 70)
*Anemic while pregnant*					
No	19	ref	ref	ref	ref
Yes	8	–0.1 (–0.4, 0.3)	–7 (–32, 29)	0.1 (–0.4, 0.5)	5 (–29, 54)
*Use of filter during pregnancy*					
Yes	14	ref	ref	ref	ref
No	14	0.1 (–0.2, 0.4)	6 (–20, 42)	0.2 (–0.2, 0.5)	22 (–13, 70)
*Ever use formula*^a,b^					
No	7			ref	ref
Yes	21			0.6 (0.2, 0.9)	79 (2*7*, 152)
*Predominant source of milk during infancy*					
Infant formula^c^	11			ref	ref
Breastmilk^d^	11			–0.5 (–0.9, –0.1)	–39 (–57, –13)
Mix of breastmilk and formula^e^	6			–0.1 (–0.6, 0.3)	–14 (–44, 32)

^a^Prenatal correlate of breastfeeding and formula variables not analyzed since correlates occur after tooth Ba was measured.

^b^Applies to index (participating) child only.

^c^Predominantly formula fed defined as maternal report of being breastfed less than 6 months and consuming ≥50% formula during first year of life.

^d^Predominantly breastfed defined as maternal report of being breastfed longer than 6 months and consuming less than 50% formula during first year of life.

^e^Mix of breastmilk and formula defined as maternal report of being breastfed for less than 6 months and consuming less than 50% formula during first year of life OR maternal report of being breastfed for longer than 6 months and consuming ≥50% formula during first year of life.

**Table 4:** Correlates of log-transformed prenatal and postnatal dentine Mn levels (as ^55^Mn:^43^Ca) from sex-adjusted linear mixed models.

		Prenatal Mn		Postnatal Mn	
	*N*	*β* (95% CI)	% Difference (95% CI)	*β* (95% CI)	% Difference (95% CI)
*Child sex*					
Male	18	ref	ref	ref	ref
Female	10	0.1 (–0.2, 0.4)	13 (–16, 52)	0.8 (0.1, 1.5)	122 (17, 321)
*Child age, continuous (years)*	28	–0.1 (–0.1, –0.02)	–8 (–13, –2)	–0.1 (0.3, 0.04)	–11 (–23, 3)
*Birth order*					
Second or higher	15	ref	ref	ref	ref
First	13	0.3 (–0.1, 0.6)	39 (–4, 85)	0.3 (–0.5, 1.0)	30 (–34, 156)
*Tooth type*					
Incisor	24	ref	ref	ref	ref
Molar	4	–0.4 (–0.8, –0.02)	–34 (–55, –4)	0.04 (–0.5, 0.6)	5 (–36, 70)
*Anemic while pregnant*					
No	19	ref	ref	ref	ref
Yes	8	0.3 (–0.1, 0.6)	31 (–5, 80)	–0.2 (–0.9, 0.6)	–15 (–59, 75)
*Use of filter during pregnancy*					
Yes	14	ref	ref	ref	ref
No	14	0.2 (–0.1, 0.5)	20 (–9, 59)	0.3 (–0.4, 1.0)	37 (–30, 168)
*Ever use formula*^a,b^					
No	7			ref	ref
Yes	21			–0.1 (–0.9, 0.7)	–11 (–57, 88)
*Predominant source of milk during infancy*					
Infant formula^c^	11			ref	ref
Breastmilk^d^	11			0.3 (–0.5, 1.0)	29 (–38, 170)
Mix of breastmilk and formula^e^	6			0.3 (–0.6, 1.2)	34 (–44, 222)

^a^Prenatal correlate of breastfeeding and formula variables not analyzed since correlates occur after tooth Mn was measured.

^b^Applies to index (participating) child only.

^c^Predominantly formula fed defined as maternal report of being breastfed less than 6 months and consuming ≥50% formula during first year of life.

^d^Predominantly breastfed defined as maternal report of being breastfed longer than 6 months and consuming less than 50% formula during first year of life.

^e^Mix of breastmilk and formula defined as maternal report of being breastfed for less than 6 months and consuming less than 50% formula during first year of life OR maternal report of being breastfed for longer than 6 months and consuming ≥50% formula during first year of life.

**Table 5:** Correlates of log-transformed prenatal and postnatal dentine Pb levels (as ^208^Pb:^43^Ca) from sex-adjusted linear mixed models.

		Prenatal Pb		Postnatal Pb	
	*N*	*β* (95% CI)	% Difference (95% CI)	*β* (95% CI)	% Difference (95% CI)
*Child sex*					
Male	18	ref	ref	ref	ref
Female	10	0.3 (–0.1, 0.8)	40 (–9, 117)	0.5 (–0.1, 1.1)	60 (–8, 178)
*Child age, continuous (years)*	28	0.001 (–0.1, 0.1)	–0.1 (–9, 10)	–0.05 (–0.2, 0.1)	–5 (–16, 8)
*Birth order*					
Second or higher	15	ref	ref	ref	ref
First	13	–0.1 (–0.6, 0.4)	–10 (–43, 42)	0.1 (–0.7, 0.5)	9 (–50, 65)
*Tooth type*					
Incisor	24	ref	ref	ref	ref
Molars	4	0.1 (–0.6, 0.7)	7 (–42, 99)	0.03 (–0.5, 1.1)	36 (–38, 197)
*Anemic while pregnant*					
No	19	ref	ref	ref	ref
Yes	8	0.3 (–0.1, 0.8)	41 (–11, 125)	0.5 (–0.2, 1.1)	57 (–13, 185)
*Use of filter during pregnancy*					
Yes	14	ref	ref	ref	ref
No	14	0.02 (–0.4, 0.5)	2 (–34, 57)	0.1 (–0.5, 0.7)	7 (–39, 87)
*Ever use formula*^a,b^					
No	7			ref	ref
Yes	21			–0.03 (–0.7, 0.7)	–3 (–49, 87)
*Predominant source of milk during infancy*					
Infant formula^c^	11			ref	ref
Breastmilk^d^	11			0.1 (–0.6, 0.7)	10 (–41, 104)
Mix of breastmilk and formula^e^	6			0.6 (–0.2, 1.3)	74 (–16, 264)

^a^Prenatal correlate of breastfeeding and formula variables not analyzed since correlates occur after tooth Pb was measured.

^b^Applies to index (participating) child only.

^c^Predominantly formula fed defined as maternal report of being breastfed less than 6 months and consuming ≥50% formula during first year of life.

^d^Predominantly breastfed defined as maternal report of being breastfed longer than 6 months and consuming less than 50% formula during first year of life.

^e^Mix of breastmilk and formula defined as maternal report of being breastfed for less than 6 months and consuming less than 50% formula during first year of life OR maternal report of being breastfed for longer than 6 months and consuming ≥50% formula during first year of life.

All statistical analyses were conducted using R 3.5.2 and SAS 9.4 (SAS Institute Inc.).

## Results

### Participant characteristics

A total of 28 children with complete questionnaire information and tooth metals data were included in the final analysis. Participating mothers were mostly white (93%) and had lived in the same home in Holliston, MA, since pregnancy (82%) (Table 1). Of the 26 children with siblings, nearly half were first-born (*n* = 11; 42%). About one-third of children required special services at school (*n* = 9, 32%), nearly all of whom had an individualized education plan (*n* = 8). Most mothers resided in homes serviced with water from a public drinking water (groundwater) source during pregnancy (89%) (Table 2). Fifty percent of participants had some type of water filtration system in their homes, including point of use (e.g., sink attachment or pitcher filter) and point of entry (e.g., whole house filter) systems.

### Prenatal and early postnatal values of dentine metals

**Fig. 1:**
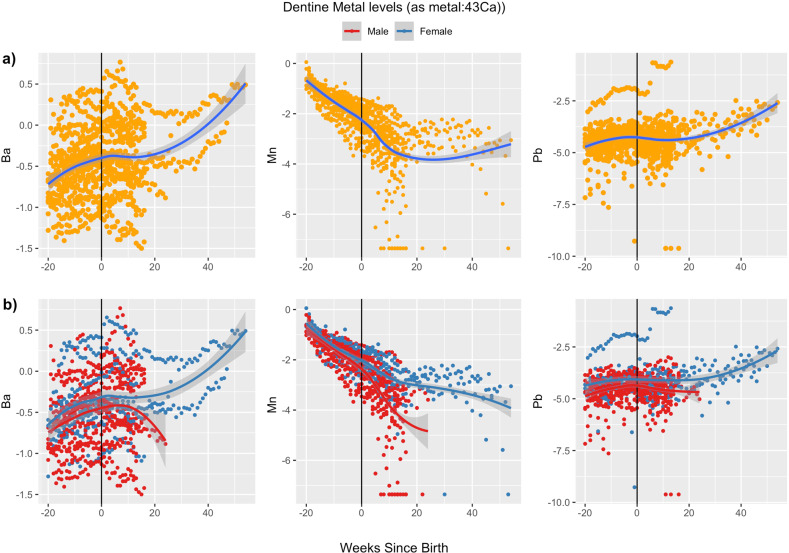
Dentine Ba, Mn, and Pb metal levels from early second trimester (20 weeks before birth) until 1 year of age (52 weeks), as ln metal:^43^Ca ratio. Dots represent individual dentine metal measurements for 28 participants, with a range of 19–70 measurements per participant. Vertical line at week 0 indicates birth. Values below the LOD were imputed as minimum measured value/sqrt(2). Line represents Loess smoother. **a**) Dentine metal levels for all participants; **b**) Dentine metal levels stratified by child sex.

We measured 12 metals in dentine but focus results on Ba, Mn, and Pb, given the historical evidence of Mn in Holliston drinking water [15], the known neurotoxicity of Mn and Pb, the interests of community members, and prior findings of tooth Ba as a dietary marker [19]. The total number of measurements of dentine metals values per participant varied, with a mean (range) of 36 (19–70) measurements. Levels and ranges of dentine Ba remained similar between the prenatal and postnatal period (Fig. 1). In the prenatal period, median (25th, 75th percentile) dentine Ba (as ^138^Ba:^43^Ca) was 0.62 (0.49, 0.71), and in the early postnatal period, the median was 0.64 (0.53, 0.94) (Supplementary Table 2). Levels of dentine Mn were highest in the early prenatal period (second trimester) and declined steadily until birth, with a slower decline and increased variability in the postnatal period. In the prenatal period, median (25th, 75th percentile) dentine Mn (as ^55^Mn:^43^Ca) was 0.25 (0.19, 0.29), while in the early postnatal period, the median was 0.06 (0.04, 0.09). Like Ba, ranges of dentine Pb remained similar between prenatal and postnatal periods. In the prenatal period, median (25th, 75th percentile) dentine Pb (as ^208^Pb:^43^Ca) was 0.01 (0.01, 0.02), and in the early postnatal period, the median was 0.01 (0.01, 0.02).

### Correlates of dentine metals values

**Fig. 2:**
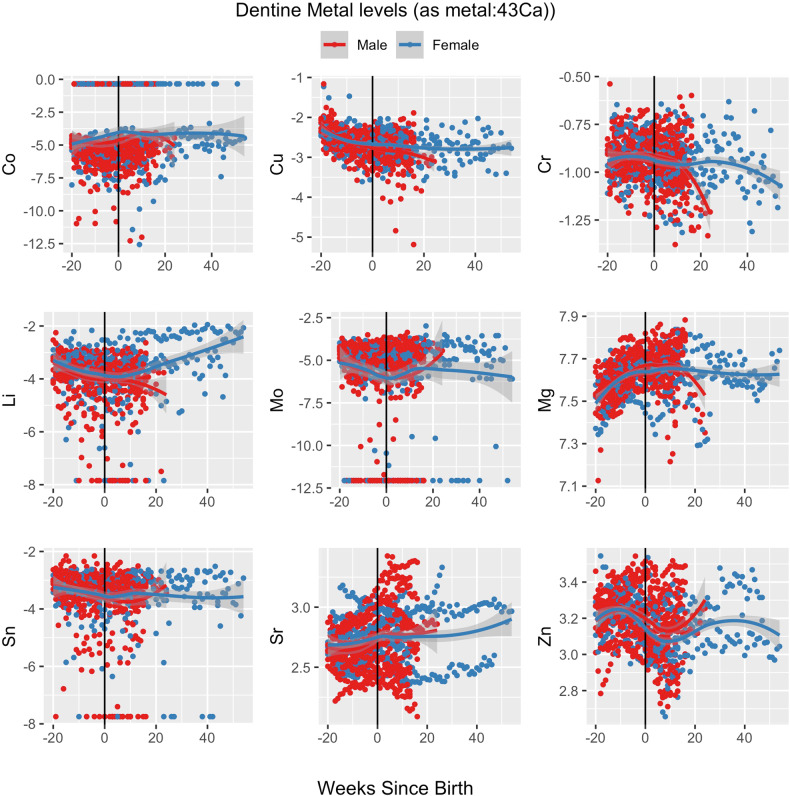
Dentine metal levels as ln metal:^43^Ca ratio from early second trimester (20 weeks before birth) until 1 year of age (52 weeks after birth), stratified by child sex. Dots represent individual dentine metal measurements for 28 participants, with a range of 19–70 measurements per participant. Vertical line at week 0 indicates birth. Values below the LOD were imputed as minimum measured value/sqrt(2). Line represents Loess smoother.

Direction and magnitude of associations between correlates and dentine metals values varied by metal. Being fed predominantly breastmilk, compared to predominantly infant formula, was associated with 39% (95% CI: –57%, –13%) lower levels of dentine Ba [*ß* = –0.5 (95% CI: –0.9, –0.1)]; being fed a mix of breastmilk and formula, compared to predominantly formula, was associated with 14% (–44%, 32%) lower dentine Ba levels [*ß* = –0.1 (95% CI: –0.6, 0.3)] (Table 3). Similarly, ever use of formula, compared to never use, was associated with higher dentine Ba (%D: 79%; 95% CI: 27%, 152%). For dentine Mn, child sex was the strongest correlate: female sex was associated with 13% (95% CI: –16%, 52%) higher prenatal and 122% (17%, 321%) higher postnatal tooth Mn values, compared to males (Table 4). Females, compared to males, also had higher levels of Ba and Pb in both the prenatal [%D Ba: 15% (95% CI: –14%, 55%); %D Pb: 40% (95% CI: –9%, 117%)] and postnatal [%D Ba: 13% (95% CI: –21%, 60%); %D Pb: 60% (95% CI: –8%, 178%)] periods (Table 5), though estimates were imprecise. Associations between other correlates and dentine Ba, Mn, and Pb were weaker and less precise (Tables 3–5; Supplementary Tables 3 and 4). Time trends in dentine metals values also appeared to differ by sex for Li and Zn, while values of Co, Cr, Cu, Mo, Mg, and Sn differed little by sex and were less variable over time (Fig. 2 and Supplementary Table 2). Correlates were not appreciably associated with other metals (Co, Cr, Cu, Li, Mo, Mg, Sn, Sr, Zn) (Supplementary Tables 5–13). There were no appreciable differences in findings from the truncated data set compared to the full data set; thus, we included all data in main analyses (Supplementary Table 14).

## Discussion

In this community-initiated pilot study, we characterized retrospective exposure to multiple metals during critical developmental periods using deciduous teeth as a biomarker of prenatal and postnatal exposure. This effort built off our previous work to address community concerns about historical exposures to neurotoxic metals [15]. These data demonstrate the feasibility of employing naturally shed teeth to understand past exposures in a community with known exposure through drinking water, but where retrospective exposure information on an individual level was otherwise not obtainable. Temporal trends of dentine metal values differed by metal. Dentine Mn levels decreased from the second trimester until birth whereas dentine Ba slightly increased and Pb remained more constant throughout the prenatal period and early life. We also found that dentine Ba may reflect dietary practices related to milk consumption during early life and that dentine levels of both Pb and Mn differ by child sex, with females having higher levels than males, although estimates were imprecise.

The observed decrease in dentine Mn from the second trimester to the first year of life has been reported in other studies and is likely related in part to the physiologic need for Mn as an essential nutrient to support healthy growth in early development [20–22]. The prenatal period is a time of rapid growth and development of the fetus, during which the demand for Mn may be greatest [21]. This is compatible with previous studies that have reported increases in maternal blood Mn during pregnancy [23–25], which supports the notion of a biological role of Mn during gestation. During gestation, exposure to Mn is more tightly regulated as the placenta may protect the fetus from direct effects of Mn overexposure [26], whereas after birth, the placenta no longer regulates Mn levels that are transported to the fetus. While development continues, the relative demand for Mn may be lower than in the prenatal period. The observed increase in variability in the postnatal period may be related to both differential exposures in the postnatal versus prenatal environments, such as direct exposure to Mn in drinking water, as well as biological changes, such as varied absorption of Mn in the gut in the postnatal period compared to the prenatal period [27, 28].

In this study, Ba levels increased slightly from the prenatal to postnatal periods. An increase in Ba levels after birth is expected, as transfer of Ba from mother to fetus is restricted by the placenta in the prenatal period [27], but after birth, increasing Ba levels likely reflect consumption of breastmilk and/or formula [19]. One previous study found that ^138^Ba:^43^Ca distributions in teeth accurately reflect the dietary transition from mother’s milk to other sources during the weaning process and concluded that ^138^Ba:^43^Ca ratios in teeth could be used to assess dietary transitions from predominantly breastmilk to predominantly infant formula or solid food intake [19]. In that study, concentrations of ^138^Ba:^43^Ca were higher in individuals who reported formula use compared to breastfeeding [19], which is consistent with our findings. While there is limited evidence that Ba is neurotoxic, toxicological evidence has shown that Ba acts as a competitive potassium channel antagonist leading to decreased potassium in blood plasma [29]. Given that many metals have been shown to cause increased intracellular Ca [30, 31], and Ca-gated potassium channels regulate intracellular Ca levels [32], Ba may play an important role in the joint or interactive effects of multiple metals.

The use of deciduous teeth as a biomarker of exposure is a novel technique that is rapidly evolving; as such, few studies have measured metals with similar fine-scale resolution and with the same analytic technique as in our study. Using externally corrected values, we were able to compare trends between the ELEMENT cohort, a prospective birth cohort in Mexico City where no specific environmental source of Mn exposure was identified, and our ACHIEVE study [21]. Dentine Mn levels and trends over time were similar between the two cohorts. Furthermore, few studies have examined correlates of metals in early life [21, 33–38], and of those, only a handful have looked at correlates of tooth metals [21, 37, 38], given the novelty of the tooth biomarker. In the Center for the Health Assessment of Mothers and Children of Salinas (CHAMACOS) cohort in Salinas Valley, CA, authors found that application of Mn-containing fungicides, take-home occupational variables (e.g., storing farm shoes inside the home), and maternal smoking were associated with higher levels of tooth Mn levels in the prenatal period [37]. In an urban Los Angeles, CA community residing near a battery smelter, maternal education, child sex, proximity to freeways, breastfeeding practices, smoking, and working in a Pb-exposed occupation were associated with soil and tooth levels of Pb in early life [38]. In the ELEMENT study, mother’s age at birth was associated with dentine Pb, but not dentine Mn, while child’s sex, maternal education, and maternal report of smoking were not associated with either Mn or Pb levels [21].

In our data, the levels of Mn and Pb tooth levels varied by child sex and this difference was more prominent in the postnatal period. There is both epidemiological and toxicological evidence suggesting that females, compared to males, may have increased Mn absorption [28, 39–41]. Furthermore, a recent study of US adults reported significantly higher blood Mn levels in females compared to males [40], suggesting there may be potential differences in Mn absorption or metabolism between females and males. One hypothesis for the difference between females and males may be related to iron (Fe) status because women are more likely to be iron deficient [28, 42, 43]. Mn and Fe share common absorption and transport pathways [44, 45] and increased Mn absorption may be due to lower Fe levels among females and/or upregulation of these shared mechanisms of Mn and Fe gastrointestinal uptake [46, 47]. Whether similar mechanisms are at play among infants to impact dentine Mn levels is unknown.

Our pilot study demonstrates the feasibility of obtaining retrospective data on a large panel of metals to address community concerns about past exposures. The tooth biomarker is particularly useful in settings where past exposures that occurred in early life are of interest and shed baby teeth are readily available. On the other hand, the tooth biomarker is less useful in scenarios that require immediate exposure information, such as crisis management, and/or where children are too young to shed teeth. As a pilot study, the sample size is small; therefore, there is limited statistical power to detect associations between correlates and tooth metals. However, up to 70 measurements of metal values were obtained from each tooth, yielding repeated outcome measurements for each participant, which increases statistical power. Our study also demonstrates that teeth allow for the direct examination of fetal and infant metal levels, rather than using surrogates of prenatal exposure (e.g., maternal blood) or biomarkers of current or recent childhood exposure (e.g., hair, toenails, blood). While it is unclear which portion of dentine metal levels may reflect endogenous versus exogenous sources, previous epidemiologic studies have found correlations between metals in environmental matrices and dentine levels [37, 38, 48]. Furthermore, a study in rats found that tooth Mn levels were strongly correlated with oral dose of Mn as well as with blood and brain Mn [16], suggesting that teeth capture exogenous oral exposure to Mn. The rate of special services required at school was high among participants of this pilot study (32.1%) compared to the US national rate (14.0%) [49] and to the rate within Holliston for the 2020–2021 academic year (20.6%) [50]. It is possible that parents who suspected that their children were exposed to metals in early life and whose children require special services in school were more likely to enroll in this study than parents of children without special services. However, given that tooth metal values were unknown at the time of enrollment and that we did not estimate associations between metals and use of special services, selection bias should not impact findings of the current study. We also lack information on potential key covariates including duration of pregnancy (i.e., gestational age) and other dietary sources. In addition, there is potential for misclassification of this self-reported covariate information, though this misclassification is likely to be nondifferential with respect to tooth metal levels because participants were unaware of their children’s tooth metal levels.

Public health recommendations to decrease exposure to environmental contaminants are often targeted toward protecting the most vulnerable (i.e., those more likely to be exposed) and susceptible (i.e., those more likely to experience an adverse effect) populations. Although it is well documented that infants and children are both vulnerable and susceptible to toxic exposures [1, 2, 5], understanding the precise timing of toxic exposures that lead to greater adverse effects will better inform interventions to decrease exposure during critical developmental windows. In this community-initiated pilot study, deciduous teeth provided the ability to assess exposure retrospectively and noninvasively to multiple metals with high temporal resolution. Deciduous teeth may serve as a useful tool for communities that have experienced historic environmental exposures.

## Supplementary information

Supplementary Material
