# Supplementary material for: Multiple metals in children’s deciduous teeth: results from a community-initiated pilot study
Source: J Expo Sci Environ Epidemiol. 2021 Nov 8;32(3):408–17. doi: 10.1038/s41370-021-00400-x (PMC9079191; doi:10.1038/s41370-021-00400-x)
Supplement: Supplementary file 1 — Supplementary Material [file 41370_2021_400_MOESM1_ESM.docx]

**SUPPLEMENTAL MATERIAL**

**TITLE:** Characterizing metals exposure during critical periods of development using deciduous teeth in a community-initiated pilot study

Alexa Friedman^1^, Julia A. Bauer^1,2^, Christine Austin^3^, Timothy J. Downs^4^, Yorghos Tripodis^5^, Wendy Heiger-Bernays^1^, Roberta F. White^1,6^, Manish Arora^3^, Birgit Claus Henn^1^

**Author Affiliations:**

^1^ Department of Environmental Health, Boston University School of Public Health, Boston, MA, USA

^2^ Department of Epidemiology, Geisel School of Medicine, Dartmouth College, Lebanon, NH, USA

^3^ Department of Environmental Medicine and Public Health, Icahn School of Medicine at Mount Sinai, New York, NY, USA

^4^ Department of International Development, Community, and Environment, Clark University, Worcester, MA, USA

^5^ Department of Biostatistics, Boston University School of Public Health, MA, USA

^6^ Department of Neurology, Boston University School of Medicine, Boston, MA, USA

Number of Tables: 14

Number of Figures:

Table S1: Limit of detection and detection frequencies for all metals included in analysis

Table S2: Prenatal and postnatal dentine metal levels by child sex (as ratio of metal: ^43^Ca)

Table S3: Additional correlates of log-transformed prenatal dentine, Ba, Mn and Pb levels from sex-adjusted linear mixed models

Table S4: Additional correlates of log-transformed postnatal dentine, Ba, Mn and Pb levels from sex-adjusted linear mixed models

Table S5: Correlates of log-transformed prenatal and postnatal dentine Co levels (as ^59^Co^43^Ca) from sex-adjusted linear mixed models

Table S6: Correlates of log-transformed prenatal and postnatal dentine Cr levels (as ^52^Cr^43^Ca) from sex-adjusted linear mixed models

Table S7: Correlates of log-transformed prenatal and postnatal dentine Cu levels (as ^63^Cu:^43^Ca) from sex-adjusted linear mixed models

Table S8: Correlates of log-transformed prenatal and postnatal dentine Li levels (as ^7^Li:^43^Ca) from sex-adjusted linear mixed models

Table S9: Correlates of log-transformed prenatal and postnatal dentine Mg levels (as ^25^Mg^43^Ca) from sex-adjusted linear mixed models

Table S10: Correlates of log-transformed prenatal and postnatal dentine Mo levels (as ^95^Mo^43^Ca) from sex-adjusted linear mixed models

Table S11: Correlates of log-transformed prenatal and postnatal dentine Sr levels (as ^118^Sn^43^Ca) from sex-adjusted linear mixed models

Table S12: Correlates of log-transformed prenatal and postnatal dentine Sr levels (as ^88^Sr^43^Ca) from sex-adjusted linear mixed models

Table S13: Correlates of log-transformed prenatal and postnatal dentine Zn levels (as ^66^Zn^43^Ca) from sex-adjusted linear mixed models

Table S14: Correlates of log-transformed postnatal dentine, truncated to 20 weeks, Ba, Mn and Pb levels from sex-adjusted linear mixed models

| **Table S1.** Limit of detection and detection frequencies for all metals included in analysis | | |
| --- | --- | --- |
| **Metal** | **Range (min, max) of metal concentrations (as metal:ion)** | **Detection Frequency** |
| Ba | (0.22, 2.15) | 100% |
| Co | (3.5E-6, 3.5E-2) | 78% |
| Cu | (0.01, 0.31) | >99% |
| Cr | (0.18, 0.58) | 100% |
| Li | (6.0E-4, 1.4E-1) | 98% |
| Mo | (8.1E-6 - 5.1E-2) | 89% |
| Mn | (9.0E-4, 1.1) | 98% |
| Mg | (1.2E+3, 3.7E+3) | 100% |
| Pb | (9.5E-5, 5.3E-1) | >99% |
| Sn | (6.0E-4, 1.2E-1) | 97% |
| Sr | (8.1, - 30.8) | 100% |
| Zn | (14.2, 34.6) | 100% |

| **Table S2.** Prenatal and postnatal dentine metal levels by child sex (as ratio of metal: ^43^Ca)^†^ | | | | |
| --- | --- | --- | --- | --- |
|  | Prenatal | | Postnatal | |
|  | Median | (25^th^ ,75^th^) | Median | (25^th^ ,75^th^) |
| Ba | 6.2E-1 | 4.9E-1, 7.1E-1 | 6.4E-1 | 5.3E-1, 9.4E-1 |
| Male | 6.2E-1 | 4.2E-1, 6.9E-1 | 6.3E-1 | 4.9E-1, 9.3E-1 |
| Female | 5.9E-1 | 5.4E-1, 7.5E-1 | 6.5E-1 | 6.3E-1, 8.9E-1 |
| Co | 4.4E-2 | 7.1E-3, 2.1E-1 | 2.1E-1 | 5.7E-2, 3.0E-1 |
| Male | 4.4E-2 | 6.4E-3, 2.1E-1 | 1.7E-1 | 5.5E-2, 3.0E-1 |
| Female | 6.7E-2 | 8.5E-4, 3.0E-1 | 2.4E-1 | 7.4E-2, 3.0E-1 |
| Cu | 8.0E-2 | 7.3E-2, 8.6E-2 | 6.4E-2 | 5.8E-2, 7.6E-2 |
| Male | 8.2E-2 | 7.4E-2, 8.5E-2 | 6.2E-2 | 5.3E-2, 7.1E-2 |
| Female | 7.7E-2 | 7.3E-2, 8.5E-2 | 7.2E-2 | 6.4E-2, 7.6E-2 |
| Cr | 4.0E-1 | 3.9E-1, 4.2E-1 | 3.9E-1 | 3.7E-1, 4.2E-1 |
| Male | 4.0E-1 | 3.9E-1, 4.2E-1 | 4.0E-1 | 3.8E-1, 4.2E-1 |
| Female | 3.9E-1 | 3.8E-1, 4.1E-1 | 3.8E-1 | 3.7E-1, 4.1E-1 |
| Li | 3.0E-2 | 2.3E-2, 3.5E-2 | 2.4E-2 | 1.8E-2, 3.0E-2 |
| Male | 2.9E-2 | 2.3E-2, 3.4E-2 | 2.4E-2 | 1.8E-2, 2.8E-2 |
| Female | 3.3E-2 | 2.3E-2, 3.7E-2 | 3.0E-2 | 1.9E-2, 3.4E-2 |
| Mo | 7.8E-3 | 5.7E-3, 1.1E-2 | 8.5E-3 | 6.0E-3, 1.0E-2 |
| Male | 8.2E-3 | 5.9E-3, 1.1E-2 | 8.2E-3 | 5.8E-3, 1.1E-2 |
| Female | 7.1E-3 | 5.6E-3, 1.1E-2 | 8.1E-3 | 6.9E-3, 1.0E-2 |
| Mn | 2.5E-1 | 1.9E-1, 2.9E-1 | 6.0E-2 | 4.3E-2, 9.2E-2 |
| Male | 2.5E-1 | 1.9E-1, 2.8E-1 | 5.0E-2 | 3.0E-2, 9.0E-2 |
| Female | 2.7E-1 | 2.1E-1, 3.3E-1 | 8.5E-2 | 6.2E-2, 1.3E-1 |
| Mg | 2.0E+3 | 1.9E+3, 2.1E+3 | 2.0E+3 | 1.9E+3, 2.1E+3 |
| Male | 2.0E+3 | 1.9E+3, 2.1E+3 | 2.1E+3 | 2.1E+3, 2.2E+3 |
| Female | 2.1E+3 | 1.9E+3, 2.1E+3 | 2.1E+3 | 2.1E+3, 2.2E+3 |
| Pb | 1.3E-2 | 1.0E-2, 1.5E-2 | 1.3E-2 | 1.1E-2, 1.7E-2 |
| Male | 1.2E-2 | 1.0E-2, 1.6E-2 | 1.3E-2 | 1.0E-2, 1.4E-2 |
| Female | 1.5E-2 | 1.2E-2, 1.5E-2 | 1.5E-2 | 1.2E-2, 1.7E-2 |
| Sn | 7.8E-3 | 5.7E-3, 1.1E-2 | 8.5E-3 | 6.0E-3, 1.1E-2 |
| Male | 8.2E-3 | 5.8E-3, 1.1E-2 | 8.6E-3 | 4.6E-3, 1.0E-2 |
| Female | 7.1E-3 | 5.6E-3, 1.1E-2 | 8.1E-3 | 6.9E-2, 1.0E-2 |
| Sr | 1.4E+1 | 1.3E+1, 1.7E+1 | 1.6E+1 | 1.3E+1, 2.0E+1 |
| Male | 1.4E+1 | 1.3E+1, 1.5E+1 | 1.6E+1 | 1.2E+1, 1.9E+1 |
| Female | 1.4E+1 | 1.3E+1, 1.7E+1 | 1.6E+1 | 1.3E+1, 1.9E+1 |
| Zn | 2.5E+1 | 2.4E+1, 2.7E+1 | 2.3E+1 | 2.0E+1, 2.5E+1 |
| Male | 2.5E+1 | 2.4E+1, 2.7E+1 | 2.3E+1 | 1.9E+1, 2.6E+1 |
| Female | 2.5E+1 | 2.4E+1, 2.6E+1 | 2.2E+1 | 2.1E+1, 2.5E+1 |
| ^†^Descriptive statistics are based on subject-specific averages for each time period | | | | |

| **Table S3:**  Additional correlates of log-transformed prenatal dentine, Ba, Mn and Pb levels from sex-adjusted linear mixed models | | | | | | | |
| --- | --- | --- | --- | --- | --- | --- | --- |
|  |  | Prenatal Ba | | Prenatal Mn | | Prenatal Pb | |
|  | N | β (95% CI) | % Difference  (95% CI) | β (95% CI) | % Difference  (95% CI) | β (95% CI) | % Difference  (95% CI) |
| Well type |  |  |  |  |  |  |  |
| Private | 3 | ref | ref | ref | ref | ref | ref |
| Public | 25 | -0.2 (-0.7, 0.3) | -17% (-48%, 32%) | -0.2 (-0.7, 0.2) | -17% (-48%, 32%) | -0.1 (-0.8, 0.7) | -5% (-52%, 88%) |
| Filtered water use for food prep during pregnancy |  |  |  |  |  |  |  |
| Always | 3 | ref | ref | ref | ref | ref | ref |
| Rarely / Sometimes | 4 | 0.3 (-0.4, 0.9) | 29% (-31%, 142%) | 0.3 (-0.4, 0.9) | 29% (-28%, 134%) | -0.5 (-1.4, 0.1) | -39% (-75%, 49%) |
| Never | 21 | 0.2 (-0.3, 0.7) | 18% (-28%, 91%) | -0.1 (-0.6, 0.3) | -14% (-45%, 37%) | -0.5 (-1.2, 0.2) | -39% (-70%, 20%) |
| Filtered water use for coffee/tea prep during pregnancy |  |  |  |  |  |  |  |
| Always | 6 | ref | ref | ref | ref | ref | ref |
| Rarely / Sometimes | 6 | 0.4 (-0.1, 0.9) | 53% (-2%, 138%) | 0.2 (-0.3, 0.6) | 17% (-27%, 87%) | -0.01 (-0.7, 0.7) | -1% (-50%, 102%) |
| Never | 16 | 0.1 (-0.3, 0.4) | 9% (-23%, 53%) | -0.1 (-0.5, 0.3) | -8% (-36%, 33%) | -0.1 (-0.7, 0.4) | -14% (-50%, 48%) |
| Bottled water use during pregnancy |  |  |  |  |  |  |  |
| Always | 2 | ref | ref | ref | ref | ref | ref |
| Rarely / Sometimes | 17 | 0.2 (-0.5, 0.8) | 21% (-34%, 122%) | -0.4 (-1.0, 0.3) | -31% (-62%, 26%) | 0.5 (-0.5, 1.4) | 58% (-34%, 280%) |
| Never | 9 | 0.1 (-0.6, 0.8) | -8% (-45%, 112%) | -0.5 (-1.2, 0.2) | -37% (-67%, 22%) | 0.5 (-0.6, 1.5) | 59% (-40%, 319%) |

| **Table S4:**  Additional correlates of log-transformed postnatal dentine, Ba, Mn and Pb levels from sex-adjusted linear mixed models | | | | | | | |
| --- | --- | --- | --- | --- | --- | --- | --- |
|  |  | Postnatal Ba | | Postnatal Mn | | Postnatal Pb | |
|  | N | β (95% CI) | % Difference  (95% CI) | β (95% CI) | % Difference  (95% CI) | β (95% CI) | % Difference  (95% CI) |
| Well type |  |  |  |  |  |  |  |
| Private | 3 | ref | ref | ref | ref | ref | ref |
| Public | 25 | -0.3 (-0.9, 0.3) | -25% (-56%, 29%) | 0.1 (-0.9, 1.2) | 13% (-59%, 210%) | -0.1 (-1.0, 0.9) | -7% (-61%, 124%) |
| Filtered water use for food prep during pregnancy |  |  |  |  |  |  |  |
| Always | 3 | ref | ref | ref | ref | ref | ref |
| Rarely / Sometimes | 4 | 0.2 (-0.6, 1.0) | 25% (-41%, 163%) | 0.3 (-1.1, 1.7) | 33% (-65%, 402%) | -1.0 (-2.2, 0.1) | -64% (-88%, 6%) |
| Never | 21 | 0.2 (-0.5, 0.8) | 17% (-35%, 108%) | -0.2 (-1.4, 0.8) | -27% (-74%, 103%) | -1.0 (-1.8, -0.1) | -62% (-84%, 12%) |
| Filtered water use for coffee/tea prep during pregnancy |  |  |  |  |  |  |  |
| Always | 6 | ref | ref | ref | ref | ref | ref |
| Rarely / Sometimes | 6 | 0.5 (-0.1, 1.0) | 62% (-3%, 172%) | 0.3 (-0.8, 1.4) | 35% (-52%, 273%) | -0.2 (-1.1, 0.7) | -17% (-66%, 102%) |
| Never | 16 | -0.04 (-0.5, 0.4) | -2% (-35%, 46%) | 0.1 (-1.0, 0.7) | 14% (-61%, 91%) | -0.4 (-1.1, 0.4) | -30% (-65%, 39%) |
| Bottled water use during pregnancy |  |  |  |  |  |  |  |
| Always | 2 | ref | ref | ref | ref | ref | ref |
| Rarely / Sometimes | 17 | 0.2 (-0.6, 0.9) | 20% (-42%, 146%) | -0.2 (-1.6, 1.2) | -21% (79%, 200%) | -0.4 (-0.9, 1.6) | -43% (-55%, 351%) |
| Never | 9 | 0.01 (-0.8, 0.9) | 2% (-54%, 126%) | 0.5 (-2.0, 1.1) | -37% (-86%, 172%) | 0.2 (-1.2, 1.5) | 19% (-67%, 322%) |
| Ever breastfed† |  |  |  |  |  |  |  |
| No | 3 | ref | ref | ref | ref | ref | ref |
| Yes | 25 | -0.6 (-1.1, -0.1) | -47% (-69%, -11%) | 0.6 (-0.5, 1.7) | 86% (-33%, 416%) | 0.1 (-0.9, 1.0) | 9% (-56%, 171%) |
| † Applies to index (participating) child only | | | | | | | |

| **Table S5:** Correlates of log-transformed prenatal and postnatal dentine Co levels (as ^59^Co^43^Ca) from sex-adjusted linear mixed models | | | | | |
| --- | --- | --- | --- | --- | --- |
|  |  | Prenatal Co | | Postnatal Co | |
|  | N | β (95% CI) | % Difference  (95% CI) | β (95% CI) | % Difference  (95% CI) |
| Child sex |  |  |  |  |  |
| Male | 18 | ref | ref | ref | ref |
| Female | 10 | 0.3 (-0.6, 1.2) | 33% (-43%, 209%) | 0.1 (-0.7, 0.9) | 8% (50%, 131%) |
| Child age, continuous (years) | - | 0.1 (-0.1, 0.2) | 5% (-12%, 26%) | -0.1 (-0.3, 0.1) | -8% (-22%, 8%) |
| Birth order |  |  |  |  |  |
| Second or higher | 15 | ref | ref | ref | ref |
| First | 13 | 0.2 (-0.6, 1.1) | 123% (-46%, 177%) | 0.5 (-0.3, 1.4) | 69% (-25%, 279%) |
| Well type |  |  |  |  |  |
| Private | 3 | ref | ref | ref | ref |
| Public | 25 | 1.1 (-0.2, 2.4) | 207% (-12%, 947%) | 0.9 (-0.3, 2.1) | 135% (-25%, 639%) |
| Tooth type |  |  |  |  |  |
| Incisor | 24 | ref | ref | ref | ref |
| Molar | 4 | 0.4 (-0.9, 1.6) | 43% (-55%, 356%) | 0.1 (-0.9, 1.1) | 12% (-58%, 197%) |
| Anemic while pregnant |  |  |  |  |  |
| No | 19 | ref | ref | ref | ref |
| Yes | 8 | -0.5 (-1.5, 0.5) | -37% (-75%, 61%) | 0.03 (-0.9, 1.0) | 3% (-57%, 148%) |
| Use of filter during pregnancy |  |  |  |  |  |
| Yes | 14 | ref | ref | ref | ref |
| No | 14 | -0.7 (-1.5, 0.1) | -51% (-77%, 5%) | -0.2 (-0.9, 0.6) | -15% (-59%, 79%) |
| Filtered water use for food prep |  |  |  |  |  |
| Always | 3 | ref | ref | ref | ref |
| Rarely / Sometimes | 4 | 0.2 (-1.6, 2.0) | 25% (-77%, 591%) | 0.3 (-1.4, 2.1) | 39% (74%, 642%) |
| Never | 21 | 0.9 (-0.5, 2.2) | 134% (-37%, -777%) | 0.1 (-1.3, 1.5) | 11% (-70%, 307%) |
| Filtered water use for coffee/tea prep |  |  |  |  |  |
| Always | 6 | ref | ref | ref | ref |
| Rarely / Sometimes | 6 | 0.4 (-1.0, 1.7) | 42% (-60%, 404%) | 0.1 (-1.3, 1.4) | 5% (-70%, 273%) |
| Never | 16 | 1.4 (-0.8 3.7) | 167% (1%, 606%) | 0.2 (-0.8, 1.2) | 24% (-53%, 227%) |
| Bottled water use |  |  |  |  |  |
| Always | 2 | ref | ref | ref | ref |
| Rarely / Sometimes | 17 | 1.6 (-0.1, 3.3) | 398% (0.2%, 2,376%) | 0.4 (-1.3, 2.1) | 50% (-70%, 658%) |
| Never | 9 | 1.2 (-0.6, 3.1) | 59% (-92%, 3,068%) | 0.2 (-0.8, 1.2) | 14% (-81%, 585%) |
| Ever breastfed*† |  |  |  |  |  |
| No | 3 |  |  | ref | ref |
| Yes | 25 |  |  | 0.7 (-0.6, 2.0) | 93% (-44%, 568%) |
| Ever use formula*† |  |  |  |  |  |
| No | 7 |  |  | ref | ref |
| Yes | 21 |  |  | -0.7 (-1.6, 0.2) | -49% (-79%, 20%) |
| Predominant source of milk during infancy |  |  |  |  |  |
| Infant Formula^a^ | 11 |  |  | ref | ref |
| Breastmilk^b^ | 11 |  |  | 0.8 (-0.1, 1.7) | 121% (-4%, 408%) |
| Mix of Breastmilk and Formula^c^ | 6 |  |  | 0.8 (-0.2, 1.9) | 127% (-16%, 514%) |
| ^*^Prenatal correlate of breastfeeding and formula variables not analyzed since correlates occur after tooth Co was measured  † Applies to index (participating) child only  ^a^ Predominantly formula fed defined as maternal report of being breastfed less than 6 months & consuming > 50% formula during first year of life  ^b^ Predominantly breastfed defined as maternal report of being breastfed longer than 6 months & consuming less than 50% formula during first year of life  ^c^ Mix of breastmilk and formula defined as maternal report of being breastfed for less than 6 months & consuming less than 50% formula during first year of life OR maternal report of being breastfed for longer than 6 months & consuming > 50% formula during first year of life | | | | | |

| **Table S6:** Correlates of log-transformed prenatal and postnatal dentine Cr levels (as ^52^Cr^43^Ca) from sex-adjusted linear mixed models | | | | | |
| --- | --- | --- | --- | --- | --- |
|  |  | Prenatal Cr | | Postnatal Cr | |
|  | N | β (95% CI) | % Difference  (95% CI) | β (95% CI) | % Difference (95% CI) |
| Child sex |  |  |  |  |  |
| Male | 18 | ref | ref | ref | ref |
| Female | 10 | -0.004 (-0.1, 0.1) | -0.4% (-7%, 7%) | 0.002 (-0.1, 0.1) | 0% (-8%, 9%) |
| Child age, continuous (years) | - | 0.003 (-0.01, 0.01) | 0.3% (-1.2%, 1.8%) | -0.004 (-0.02, 0.01) | -0.4% (-2%, 1%) |
| Birth order |  |  |  |  |  |
| Second or higher | 15 | ref | ref | ref | ref |
| First | 13 | -0.002 (-0.1, 0.1) | -0.2% (-7%, 8%) | -0.02 (-0.1, 0.1) | -2% (-10%, 8%) |
| Well type |  |  |  |  |  |
| Private | 3 | ref | ref | ref | ref |
| Public | 25 | 0.04 (-0.1, 0.1) | 2% (-8%, 14%) | 0.02 (-0.1, 0.2) | 2% (-11%, 16%) |
| Tooth type |  |  |  |  |  |
| Incisor | 24 | ref | ref | ref | ref |
| Molar | 4 | -0.03 (-0.1, 0.1) | -3% (-11%, 6%) | -0.1 (-0.2, 0.1) | -5% (-15%, 6%) |
| Anemic while pregnant |  |  |  |  |  |
| No | 19 | ref | ref | ref | ref |
| Yes | 8 | 0.1 (-0.03, 0.1) | 6% (-2%, 13%) | 0.1 (-0.01, 0.2) | 9% (-1%, 19%) |
| Use of filter during pregnancy |  |  |  |  |  |
| Yes | 14 | ref | ref | ref | ref |
| No | 14 | 0.03 (-0.03, 0.10) | 3% (-3%, 10%) | 0.01 (-0.1, 0.1) | 1% (-7%, 10%) |
| Filtered water use for food prep |  |  |  |  |  |
| Always | 3 | ref | ref | ref | ref |
| Rarely / Sometimes | 4 | -0.03 (-0.2, 0.1) | -3% (-16%, 12%) | -0.01 (-0.2, 0.1) | -6% (-21%, 13%) |
| Never | 21 | -0.01 (-0.1, 0.1) | -1% (-12%, 11%) | -0.03 (-0.2, 0.1) | -4% (-16%, 11%) |
| Filtered water use for coffee/tea prep |  |  |  |  |  |
| Always | 6 | ref | ref | ref | ref |
| Rarely / Sometimes | 6 | -0.04 (-0.2, 0.1) | -4% (-14%, 7%) | -0.1 (-0.3, 0.02) | -11% (-22%, 1%) |
| Never | 16 | -0.04 (-0.1, 0.1) | -4% (-12%, 5%) | -0.1 (-0.2, 0.1) | -5% (-14%, 5%) |
| Bottled water use |  |  |  |  |  |
| Always | 2 | ref | Ref | ref | ref |
| Rarely / Sometimes | 17 | 0.1 (-0.1, 0.2) | 9% (-5%, 25%) | 0.02 (-0.2, 0.2) | -2% (18%, 16%) |
| Never | 9 | 0.1 (-0.1, 0.3) | 10% (-5%, 29%) | 0.04 (-0.2, 0.2) | 4% (-14%, 26%) |
| Ever breastfed*† |  |  |  |  |  |
| No | 3 |  |  | ref | ref |
| Yes | 25 |  |  | -0.1 (-0.2, 0.1) | -9% (-20%, 4%) |
| Ever use formula*† |  |  |  |  |  |
| No | 7 |  |  | ref | ref |
| Yes | 21 |  |  | -0.02 (-0.1, 0.1) | 2% (-8%, 12%) |
| Predominant source of milk during infancy |  |  |  |  |  |
| Infant Formula^a^ | 11 |  |  | ref | ref |
| Breastmilk^b^ | 11 |  |  | 0.02 (-0.1, 0.1) | 2% (-8%, 13%) |
| Mix of Breastmilk and Formula | 6 |  |  | 0.04 (-0.1, 0.2) | 4% (-7%, 17%) |
| ^*^Prenatal correlate of breastfeeding and formula variables not analyzed since correlates occur after tooth Cr was measured  † Applies to index (participating) child only  ^a^ Predominantly formula fed defined as maternal report of being breastfed less than 6 months & consuming > 50% formula during first year of life  ^b^ Predominantly breastfed defined as maternal report of being breastfed longer than 6 months & consuming less than 50% formula during first year of life  ^c^ Mix of breastmilk and formula defined as maternal report of being breastfed for less than 6 months & consuming less than 50% formula during first year of life OR maternal report of being breastfed for longer than 6 months & consuming > 50% formula during first year of life | | | | | |

| **Table S7**: Correlates of log-transformed prenatal and postnatal dentine Cu levels (as ^63^Cu:^43^Ca) from sex-adjusted linear mixed models | | | | | |
| --- | --- | --- | --- | --- | --- |
|  |  | Prenatal Cu | | Postnatal Cu | |
|  | N | β (95% CI) | % Difference (95% CI) | β (95% CI) | % Difference (95% CI) |
| Child sex |  |  |  |  |  |
| Male | 18 | ref | ref | ref | ref |
| Female | 10 | -0.03 (-0.2, 0.1) | -3% (-14%, 10%) | 0.1 (-0.1, 0.3) | 13% (-4%, 33%) |
| Child age, continuous (years) | - | -0.01 (-0.04, 0.01) | -1% (-4%, 1%) | -0.01 (-0.05, 0.03) | -1% (-5%, 3%) |
| Birth order |  |  |  |  |  |
| Second or higher | 15 | ref | ref | ref | ref |
| First | 13 | -0.01 (-0.1, 0.1) | -1% (-11%, 15%) | 0.05 (-0.1, 0.2) | 5% (-12%, 25%) |
| Well type |  |  |  |  |  |
| Private | 3 | ref | ref | ref | ref |
| Public | 25 | 0.1 (-0.2, 0.2) | 5% (-13%, 27%) | 0.05 (-0.2, 0.3) | 5% (-19%, 36%) |
| Tooth type |  |  |  |  |  |
| Incisor | 24 | ref | ref | ref | ref |
| Molar | 4 | -0.1 (-0.3, 0.1) | -9% (-22%, 7%) | -0.2 (-0.4, 0.1) | -16% (-33%, 4%) |
| Anemic while pregnant |  |  |  |  |  |
| No | 19 | ref | ref | ref | ref |
| Yes | 8 | -0.02 (-0.2, 0.1) | -3% (-15%, 11%) | -0.1 (-0.3, 0.1) | -9% (-24%, 9%) |
| Use of filter during pregnancy |  |  |  |  |  |
| Yes | 14 | ref | ref | ref | ref |
| No | 14 | 0.05 (-0.1, 0.2) | 5% (-7%, 18%) | 0.1 (-0.1, 0.2) | 5% (-11%, 24%) |
| Filtered water use for food prep |  |  |  |  |  |
| Always | 3 | ref | ref | ref | ref |
| Rarely / Sometimes | 4 | -0.02 (-0.3, 0.2) | -2% (-22%, 25%) | 0.1 (-0.3, 0.4) | 9% (-22%, 53%) |
| Never | 21 | -0.1 (-0.3, 0.1) | -13% (-27%, 5%) | -0.1 (-0.4, 0.2) | -8% (-29%, 20%) |
| Filtered water use for coffee/tea prep |  |  |  |  |  |
| Always | 6 | ref | ref | ref | ref |
| Rarely / Sometimes | 6 | -0.01 (-0.2, 0.2) | -1% (-17%, 21%) | -0.01 (-0.3, 0.3) | -1% (-23%, 31%) |
| Never | 16 | -0.1 (-0.2, 0.1) | -8% (-20%, 7%) | -0.1 (-0.3, 0.2) | -6% (-24%, 15%) |
| Bottled water use during pregnancy |  |  |  |  |  |
| Always | 2 | ref | ref | ref | ref |
| Rarely / Sometimes | 17 | 0.02 (-0.2, 0.3) | 2% (-21%, 31%) | -0.03 (-0.4, 0.4) | -0.3% (-30%, 41%) |
| Never | 9 | -0.01 (-0.3, 0.3) | -1% (-25%, 30%) | -0.03 (-0.4, 0.4) | -3% (-34%, 43%) |
| Ever breastfed*† |  |  |  |  |  |
| No | 3 |  |  | ref | ref |
| Yes | 25 |  |  | 0.1 (-0.2, 0.4) | 13% (-13%, 47%) |
| Ever use formula*† |  |  |  |  |  |
| No | 7 |  |  | ref | ref |
| Yes | 21 |  |  | -0.1 (-0.3, 0.1) | -10% (-26%, 9%) |
| Predominant source of milk during infancy |  |  |  |  |  |
| Infant Formula^a^ | 11 |  |  | ref | ref |
| Breastmilk^b^ | 11 |  |  | 0.1 (-0.1, 0.3) | 7% (-11%, 30%) |
| Mix of Breastmilk and Formula^c^ | 6 |  |  | -0.01 (-0.3, 0.2) | -1% (-21%, 24%) |
| *Prenatal correlate of breastfeeding and formula variables not analyzed since correlates occur after tooth Cu was measured  † Applies to index (participating) child only  ^a^ Predominantly formula fed defined as maternal report of being breastfed less than 6 months & consuming > 50% formula during first year of life  ^b^ Predominantly breastfed defined as maternal report of being breastfed longer than 6 months & consuming less than 50% formula during first year of life  ^c^ Mix of breastmilk and formula defined as maternal report of being breastfed for less than 6 months & consuming less than 50% formula during first year of life OR maternal report of being breastfed for longer than 6 months & consuming > 50% formula during first year of life | | | | | |

| **Table S8:** Correlates of log-transformed prenatal and postnatal dentine Li levels (as ^7^Li:^43^Ca) from sex-adjusted linear mixed models | | | | | |
| --- | --- | --- | --- | --- | --- |
|  |  | Prenatal Li | | Postnatal Li | |
|  | N | β (95% CI) | % Difference  (95% CI) | β (95% CI) | % Difference  (95% CI) |
| Child sex |  |  |  |  |  |
| Male | 18 | ref | ref | ref | ref |
| Female | 10 | 0.1 (-0.3, 0.6) | 16% (-27%, 83%) | 0.2 (-0.4, 0.9) | 23% (-34%, 129%) |
| Child age, continuous (years) | - | -0.04 (-0.1, 0.1) | 4% (-6%, 14%) | 0.05 (-0.1, 0.1) |  |
| Birth order |  |  |  |  |  |
| Second or higher | 15 | ref | ref | ref | ref |
| First | 13 | 0.2 (-0.3, 0.8) | 28% (-21%, 107%) | 0.04 (-0.7, 0.7) | 4% (-46%, 102%) |
| Well type |  |  |  |  |  |
| Private | 3 | ref | ref | ref | ref |
| Public | 25 | -0.2 (-1.0, 0.5) | -22% (-62%, 60%) | -0.04 (-1.1, 1.1) | -3% (-64%, 158%) |
| Tooth type |  |  |  |  |  |
| Incisor | 24 | ref | ref | ref | ref |
| Molar | 4 | 0.3 (-0.4, 0.9) | 34% (29%, 150%) | 0.6 (-0.3, 1.4) | 75% (-23%, 298%) |
| Anemic while pregnant |  |  |  |  |  |
| No | 19 | ref | ref | ref | ref |
| Yes | 8 | 0.1 (-0.4, 0.7) | 15% (-32%, 92%) | 0.1 (-0.6, 0.9) | 13% (-44%, 130%) |
| Use of filter during pregnancy |  |  |  |  |  |
| Yes | 14 | ref | ref | ref | ref |
| No | 14 | 0.1 (-0.3, 0.6) | 14% (-27%, 77%) | 0.3 (-0.4, 0.9) | 29% (-29%, 123%) |
| Filtered water use for food prep |  |  |  |  |  |
| Always | 3 | ref | ref | ref | ref |
| Rarely / Sometimes | 4 | 0.4 (-0.7, 1.4) | 44% (-46%, 282%) | 0.5 (-0.9, 1.9) | 60% (-57%, 499%) |
| Never | 21 | 0.2 (-0.6, 1.0) | 22% (-43%, 159%) | 0.4 (-0.7, 1.5) | 51% (-46%, 319%) |
| Filtered water use for coffee/tea prep | |  |  |  |  |
| Always | 6 | ref | ref | ref | ref |
| Rarely / Sometimes | 6 | 0.6 (-0.2, 1.3) | 77% (-13%, 259%) | 0.7 (-0.3, 1.7) | 104% (-23%, 437%) |
| Never | 16 | 0.1 (-0.5, 0.7) | 9% (-37%, 89%) | 0.2 (-0.6, 1.0) | 26% (-41%, 167%) |
| Bottled water use during pregnancy |  |  |  |  |  |
| Always | 2 | ref | ref | ref | ref |
| Rarely / Sometimes | 17 | 0.3 (-0.7, 1.2) | 30% (-49%, 232%) | 0.9 (-0.4, 2.2) | 152% (-27%, 763%) |
| Never | 9 | 0.5 (-0.6, 1.6) | 66% (-41%, 367%) | 1.2 (-0.3, 2.6) | 219% (-19%, 1,147%) |
| Ever breastfed*† |  |  |  |  |  |
| No | 3 |  |  | ref | ref |
| Yes | 25 |  |  | -0.7 (-1.7, 0.3) | -51% (-82%, 31%) |
| Ever use formula*† |  |  |  |  |  |
| No | 7 |  |  | ref | ref |
| Yes | 21 |  |  | 0.2 (-0.6, 0.9) | 17% (-43%, 142%) |
| Predominant source of milk during infancy | |  |  |  |  |
| Infant Formula^a^ | 11 |  |  | ref | ref |
| Breastmilk^b^ | 11 |  |  | 0.03 (-0.7, 0.8) | 5% (-49%, 114%) |
| Mix of Breastmilk and Formula^c^ | 6 |  |  | -0.2 (-1.1, 0.7) | -19% (-66%, 89%) |
| *Prenatal correlate of breastfeeding and formula variables not analyzed since correlates occur after tooth Li was measured  † Applies to index (participating) child only  ^a^ Predominantly formula fed defined as maternal report of being breastfed less than 6 months & consuming > 50% formula during first year of life  ^b^ Predominantly breastfed defined as maternal report of being breastfed longer than 6 months & consuming less than 50% formula during first year of life  ^c^ Mix of breastmilk and formula defined as maternal report of being breastfed for less than 6 months & consuming less than 50% formula during first year of life OR maternal report of being breastfed for longer than 6 months & consuming > 50% formula during first year of life | | | | | |

| **Table S9:** Correlates of log-transformed prenatal and postnatal dentine Mg levels (as ^25^Mg^43^Ca) from sex-adjusted linear mixed models | | | | | |
| --- | --- | --- | --- | --- | --- |
|  |  | Prenatal Mg | | Postnatal Mg | |
|  | N | β (95% CI) | % Difference  (95% CI) | β (95% CI) | % Difference  (95% CI) |
| Child sex |  |  |  |  |  |
| Male | 18 | ref | ref | ref | ref |
| Female | 10 | -0.01 (-0.1, 0.05) | -1% (-6%, 5%) | -0.04 (-0.1, 0.03) | -4% (-10%, 3%) |
| Child age, continuous (years) | - | -0.003 (-0.02, 0.01) | -0.3% (-2%, 1%) | 0.002 (-0.01, 0.02) | -0.2% (-1%, 2%) |
| Birth order |  |  |  |  |  |
| Second or higher | 15 | ref | ref | ref | ref |
| First | 13 | 0.04 (-0.1, 0.05) | -4% (-10%, 2%) | -0.02 (-0.1 0.6) | -2% (-8%, 5%) |
| Well type |  |  |  |  |  |
| Private | 3 | ref | ref | ref | ref |
| Public | 25 | 0.02 (-0.1, 0.1) | 2% (-7%,12%) | -0.04 (-0.1, 0.1) | -4% (-13%, 6%) |
| Tooth type |  |  |  |  |  |
| Incisor | 24 | ref | ref | ref | ref |
| Molar | 4 | -0.03 (-0.1, 0.1) | -3% (-10%, 5%) | -0.05 (-0.1, 0.04) | -5% (-13%, 4%) |
| Anemic while pregnant |  |  |  |  |  |
| No | 19 | ref | ref | ref | ref |
| Yes | 8 | -0.03 (-0.1, 0.03) | -3% (-10%, 3%) | -0.03 (-0.1, 0.1) | -3% (-10%, 4%) |
| Use of filter during pregnancy |  |  |  |  |  |
| Yes | 14 | ref | ref | ref | ref |
| No | 14 | 0.01 (-0.1, 0.1) | 1% (-5%, 7%) | 0.01 (-0.1, 0.1) | 1% (-5%, 8%) |
| Filtered water use for food prep |  |  |  |  |  |
| Always | 3 | ref | ref | ref | ref |
| Rarely / Sometimes | 4 | -0.1 (-0.2, 0.02) | -9% (-19%, 2%) | -0.1 (-0.2, 0.1) | -7% (-19%, 7%) |
| Never | 21 | -0.02 (-0.1, 0.1) | -2% (-10%, 8%) | -0.1 (-0.2, 0.1) | -5% (-15%, 6%) |
| Filtered water use for coffee/tea prep |  |  |  |  |  |
| Always | 6 | ref | ref | ref | Ref |
| Rarely / Sometimes | 6 | -0.01 (-0.1, 0.1) | -0.5% (-10%, 10%) | 0.02 (-0.1, 0.1) | 3% (-7, 13%) |
| Never | 16 | -0.03 (-0.1, 0.1) | -3% (-10%, 5%) | -0.1 (-0.1, 0.02) | -5% (-12%, 2%) |
| Bottled water use during pregnancy |  |  |  |  |  |
| Always | 2 | ref | ref | ref | ref |
| Rarely / Sometimes | 17 | 0.1 (0.1, 0.2) | 7% (-4%, 20%) | 0.04 (-0.1, 0.2) | 4% (-10%, 19%) |
| Never | 9 | 0.03 (-0.1, 0.2) | 3% (-10%, 17%) | 0.03 (-0.1, 0.2) | 4% (-11%, 20%) |
| Ever breastfed*† |  |  |  |  |  |
| No | 3 |  |  | ref | ref |
| Yes | 25 |  |  | -0.1 (-0.2, 0.04) | -7% (-16%, 3%) |
| Ever use formula*† |  |  |  |  |  |
| No | 7 |  |  | ref | ref |
| Yes | 21 |  |  | 0.1 (-0.03, 0.1) | 5% (-2%, 13%) |
| Predominant source of milk during infancy |  |  |  |  |  |
| Infant Formula^a^ | 11 |  |  | ref | ref |
| Breastmilk^b^ | 11 |  |  | -0.1 (-0.1, 0.03) | -7% (-13%, 0%) |
| Mix of Breastmilk and Formula^c^ | 6 |  |  | -0.02 (-0.1, 0.1) | -2% (-10%, 7%) |
| *Prenatal correlate of breastfeeding and formula variables not analyzed since correlates occur after tooth Mg was measured  † Applies to index (participating) child only  ^a^ Predominantly formula fed defined as maternal report of being breastfed less than 6 months & consuming > 50% formula during first year of life  ^b^ Predominantly breastfed defined as maternal report of being breastfed longer than 6 months & consuming less than 50% formula during first year of life  ^c^ Mix of breastmilk and formula defined as maternal report of being breastfed for less than 6 months & consuming less than 50% formula during first year of life OR maternal report of being breastfed for longer than 6 months & consuming > 50% formula during first year of life | | | | | |

| **Table S10**: Correlates of log-transformed prenatal and postnatal dentine Mo levels (as ^95^Mo^43^Ca) from sex-adjusted linear mixed models | | | | | |
| --- | --- | --- | --- | --- | --- |
|  |  | Prenatal Mo | | Postnatal Mo | |
|  | N | β (95% CI) | % Difference  (95% CI) | β (95% CI) | % Difference  (95% CI) |
| Child sex |  |  |  |  |  |
| Male | 18 | ref | ref | ref | ref |
| Female | 10 | 0.1 (-1.0, 1.2) | 9% (-63%, 220%) | 0.3 (-0.9, 1.5) | 29% (59%, 305%) |
| Child age, continuous (years) | - | 0.1 (-0.2, 0.3) | 9% (-13%, 37%) | 0.2 (-0.1, 0.4) | 18% (-7%, 49%) |
| Birth order |  |  |  |  |  |
| Second or higher | 15 | ref | ref | ref | ref |
| First | 13 | -0.8 (-1.9, 0.4) | -54% (85%, 40%) | -0.6 (-1.9, 0.6) | -47% (-84%, 79%) |
| Well type |  |  |  |  |  |
| Private | 3 | ref | ref | ref | ref |
| Public | 25 | 0.2 (-1.6, 2.0) | 22% (-78%, 562%) | -0.2 (-2.1, 1.7) | -15% (-86%, 415%) |
| Tooth type |  |  |  |  |  |
| Incisor | 24 | ref | ref | ref | ref |
| Molar | 4 | 0.1 (-1.5, 1.6) | 10% (-75%, 376%) | 0.5 (-1.1, 2.1) | 64% (-64%, 650%) |
| Anemic while pregnant |  |  |  |  |  |
| No | 19 | ref | ref | ref | ref |
| Yes | 8 | 0.4 (-0.8, 1.7) | 56% (53%, 419%) | 0.8 (-0.5, 2.1) | 124% (-37%, 699%) |
| Use of filter during pregnancy |  |  |  |  |  |
| Yes | 14 | ref | ref | ref | ref |
| No | 14 | 0.4 (-0.6, 1.5) | 53% (-45%, 326%) | 0.2 (-1.0, 1.4) | 23% (-59%, 272%) |
| Filtered water use for food prep |  |  |  |  |  |
| Always | 3 | ref | ref | ref | ref |
| Rarely / Sometimes | 4 | -1.2 (-3.5, 1.2) | 70% (97%, 184%) | -0.9 (-3.5, 1.7) | -60% (-97%, 359%) |
| Never | 21 | -0.3 (-2.2, 1.5) | 29% (-88%, 299%) | -0.1 (-2.1, 1.9) | -12% (-87%, 478%) |
| Filtered water use for coffee/tea prep |  |  |  |  |  |
| Always | 6 | ref | ref | ref | ref |
| Rarely / Sometimes | 6 | -0.9 (-2.7, 0.9) | -59% (-93%, 129%) | -0.6 (-2.6, 1.4) | -45% (-92%, 252%) |
| Never | 16 | -0.6 (-2.0, 0.8) | -44% (-85%, 110%) | -0.4 (-1.9, 1.1) | -35% (-85%, 177%) |
| Bottled water use during pregnancy |  |  |  |  |  |
| Always | 2 | ref | ref | ref | ref |
| Rarely / Sometimes | 17 | -0.1 (-2.4, 2.2) | -9% (-90%, 695%) | 0.1 (-2.3, 2.5) | 11% (89%, 973%) |
| Never | 9 | 0.7 (-1.9, 3.2) | 95% (-82%, 2042%) | 1.3 (-1.4, 3.9) | 256% (-71%, 4266%) |
| Ever breastfed*† |  |  |  |  |  |
| No | 3 |  |  | ref | ref |
| Yes | 25 |  |  | -0.6 (-2.6, 1.3) | -47%, (-92%, 245%) |
| Ever use formula*† |  |  |  |  |  |
| No | 7 |  |  | ref | ref |
| Yes | 21 |  |  | 1.6 (0.4, 2.9) | 409% (55%, 1564%) |
| Predominant source of milk during infancy |  |  |  |  |  |
| Infant Formula^a^ | 11 |  |  | ref | ref |
| Breastmilk^b^ | 11 |  |  | -1.3 (-2.6, -0.1) | -74% (-92%, -15%) |
| Mix of Breastmilk and Formula^c^ | 6 |  |  | 0.04 (-1.5, 1.5) | 4% (-75%, 326%) |
| ^*^Prenatal correlate of breastfeeding and formula variables not analyzed since correlates occur after tooth Mo was measured  † Applies to index (participating) child only  ^a^ Predominantly formula fed defined as maternal report of being breastfed less than 6 months & consuming > 50% formula during first year of life  ^b^ Predominantly breastfed defined as maternal report of being breastfed longer than 6 months & consuming less than 50% formula during first year of life  ^c^ Mix of breastmilk and formula defined as maternal report of being breastfed for less than 6 months & consuming less than 50% formula during first year of life OR maternal report of being breastfed for longer than 6 months & consuming > 50% formula during first year of life | | | | | |

| **Table S11**: Correlates of log-transformed prenatal and postnatal dentine Sn levels (as ^118^Sn^43^Ca) from sex-adjusted linear mixed models | | | | | |
| --- | --- | --- | --- | --- | --- |
|  |  | Prenatal Sn | | Postnatal Sn | |
|  | N | β (95% CI) | % Difference  (95% CI) | β (95% CI) | % Difference  (95% CI) |
| Child sex |  |  |  |  |  |
| Male | 18 | ref | ref | ref | ref |
| Female | 10 | -0.03 (-0.4, 0.4) | -2.5% (-33%, 42%) | 0.1 (-0.5, 0.8) | 16% (-37%, 112%) |
| Child age, continuous (years) | - | 0.03 (-0.1, 0.1) | 3% (-5%, 11%) | 0.1 (-0.1, 0.2) | 7% (-6%, 22%) |
| Birth order |  |  |  |  |  |
| Second or higher | 15 | ref | ref | ref | ref |
| First | 13 | -0.3 (-0.7, 0.1) | -26% (-50%, 9%) | -0.6 (-1.2, 0.1) | -44% (70%, 2%) |
| Well type |  |  |  |  |  |
| Private | 3 | ref | ref | ref | ref |
| Public | 25 | 0.1 (-0.6, 0.7) | 7% (-41%, 94%) | 0.1 (-0.9, 1.1) | 10% (-57%, 185%) |
| Tooth type |  |  |  |  |  |
| Incisor | 24 | ref | ref | ref | ref |
| Molar | 4 | 0.02 (-0.5, 0.6) | 2% (-39%, 71%) | 0.3 (-0.6, 1.1) | 33% (-41%, 198%) |
| Anemic while pregnant |  |  |  |  |  |
| No | 19 | ref | ref | ref | ref |
| Yes | 8 | 0.2 (-0.3, 0.6) | 18% (-23%, 80%) | 0.1 (-0.6, 0.9) | 16% (-42%, 130%) |
| Use of filter during pregnancy |  |  |  |  |  |
| Yes | 14 | ref | ref | ref | ref |
| No | 14 | -0.3 (-0.6, 0.1) | -23% (-46%, 9%) | -0.5 (-1.1, 0.1) | -38% (-64%, 8%) |
| Filtered water use for food prep |  |  |  |  |  |
| Always | 3 | ref | ref | ref | ref |
| Rarely / Sometimes | 4 | -0.6 (-1.4, 0.2) | -47% (-75%, 13%) | -1.1 (-2.3, 0.2) | -66% (90%, 13%) |
| Never | 21 | -0.1 (-0.8, 0.5) | -13% (-51%, 56%) | -0.2 (-1.2, 0.8) | -17% (-67%, 108%) |
| Filtered water use for coffee/tea prep |  |  |  |  |  |
| Always | 6 | ref | ref | ref | Ref |
| Rarely / Sometimes | 6 | 0.04 (-0.6, 0.7) | 4% (-42%, 89%) | -0.3 (-1.3, 0.7) | -27% (-71%, 84%) |
| Never | 16 | 0.3 (-0.2, 0.8) | 34% (-15%, 111%) | 0.3 (-0.4, 1.1) | 40% (-32%, 188%) |
| Bottled water use during pregnancy |  |  |  |  |  |
| Always | 2 | ref | ref | ref | ref |
| Rarely / Sometimes | 17 | -0.3 (-1.1, 0.5) | -24% (-65%, 62%) | -0.3 (-1.6, 1.0) | -25% (-78%, 157%) |
| Never | 9 | -0.1 (-1.0, 0.8) | 7% (-60%, 117%) | 0.1 (-1.3, 1.5) | 11% (-72%, 332%) |
| Ever breastfed*† |  |  |  |  |  |
| No | 3 |  |  | ref | ref |
| Yes | 25 |  |  | 0.2 (-0.8, 1.3) | 27% (-52%, 241%) |
| Ever use formula*† |  |  |  |  |  |
| No | 7 |  |  | ref | ref |
| Yes | 21 |  |  | 0.2 (-0.7, 0.8) | 2% (-50%, 106%) |
| Predominant source of milk during infancy |  |  |  |  |  |
| Infant Formula^a^ | 11 |  |  | ref | ref |
| Breastmilk^b^ | 11 |  |  | 0.2 (-1.0, 0.5) | -22% (-61%, 57%) |
| Mix of Breastmilk and Formula^c^ | 6 |  |  | -0.1 (1.0, 0.8) | -12% (-61%, 103%) |
| ^*^Prenatal correlate of breastfeeding and formula variables not analyzed since correlates occur after tooth Sn was measured  † Applies to index (participating) child only  ^a^ Predominantly formula fed defined as maternal report of being breastfed less than 6 months & consuming > 50% formula during first year of life  ^b^ Predominantly breastfed defined as maternal report of being breastfed longer than 6 months & consuming less than 50% formula during first year of life  ^c^ Mix of breastmilk and formula defined as maternal report of being breastfed for less than 6 months & consuming less than 50% formula during first year of life OR maternal report of being breastfed for longer than 6 months & consuming > 50% formula during first year of life | | | | | |

| **Table S12**: Correlates of log-transformed prenatal and postnatal dentine Sr levels (as ^88^Sr^43^Ca) from sex-adjusted linear mixed models | | | | | |
| --- | --- | --- | --- | --- | --- |
|  |  | Prenatal Sr | | Postnatal Sr | |
|  | N | β (95% CI) | % Difference  (95% CI) | β (95% CI) | % Difference  (95% CI) |
| Child sex |  |  |  |  |  |
| Male | 18 | ref | ref | ref | ref |
| Female | 10 | 0.04 (-0.1, 0.2) | 4% (-10%, 19%) | -0.02 (-0.2, 0.2) | -2% (-21%, 23%) |
| Child age, continuous (years) | - | 0.04 (0.01, 0.06) | 4% (1%, 6%) | 0.04 (-0.01, 0.09) | 4% (-0.3%, 9%) |
| Birth order |  |  |  |  |  |
| Second or higher | 15 | ref | ref | ref | ref |
| First | 13 | -0.1 (-0.2, 0.1) | -5% (-18%, 10%) | -0.04 (-0.3, 0.2) | -4% (-24%, 22%) |
| Well type |  |  |  |  |  |
| Private | 3 | ref | ref | ref | ref |
| Public | 25 | -0.2 (-0.4, 0.02) | -18% (-33%, 1%) | -0.4 (-0.7, -0.1) | -32% (-50%, 6%) |
| Tooth type |  |  |  |  |  |
| Incisor | 24 | ref | ref | ref | ref |
| Molar | 4 | 0.1 (-0.1, 0.3) | 15% (-4%, 39%) | 0.04 (-0.3, 0.4) | 5% (-23%, 41%) |
| Anemic while pregnant |  |  |  |  |  |
| No | 19 | ref | ref | ref | ref |
| Yes | 8 | -0.03 (-0.2, 0.1) | -3% (-18%, 13%) | 0.1 (-0.2, 0.4) | 10% (-14%, 40%) |
| Use of filter during pregnancy |  |  |  |  |  |
| Yes | 14 | ref | ref | ref | ref |
| No | 14 | 0.03 (-0.1, 0.2) | 3% (-10%, 18%) | 0.1 (-0.1, 0.3) | 13% (-8%, 39%) |
| Filtered water use for food prep |  |  |  |  |  |
| Always | 3 | ref | ref | ref | ref |
| Rarely / Sometimes | 4 | 0.1 (-0.2, 0.4) | 11% (-17%, 48%) | 0.1 (-0.3, 0.6) | 15% (-27%, 80%) |
| Never | 21 | 0.2 (-0.1, 0.4) | 18% (-6%, 47%) | 0.3 (-0.1, 0.6) | 30% (-9%, 83%) |
| Filtered water use for coffee/tea prep |  |  |  |  |  |
| Always | 6 | ref | ref | ref | ref |
| Rarely / Sometimes | 6 | 0.2 (0.02, 0.4) | 24% (0%, 53%) | 0.5 (0.1, 0.8) | 60% (18%, 116%) |
| Never | 16 | 0.1 (-0.1, 0.3) | 11% (-6%, 31%) | 0.2 (-0.1, 0.4) | 17% (-8%, 49%) |
| Bottled water use during pregnancy |  |  |  |  |  |
| Always | 2 | ref | ref | ref | ref |
| Rarely / Sometimes | 17 | 0.2 (-0.1, 0.5) | 24% (-6%, 64%) | 0.2 (-0.2, 0.7) | 24% (-21%, 94%) |
| Never | 9 | 0.2 (-0.2, 0.5) | 18% (-13%, 61%) | 0.1 (-0.4, 0.6) | 13% (-31%, 86%) |
| Ever breastfed*† |  |  |  |  |  |
| No | 3 |  |  | ref | ref |
| Yes | 25 |  |  | -0.5 (-0.8, -0.2) | -41% (-56%, -21%) |
| Ever use formula*† |  |  |  |  |  |
| No | 7 |  |  | ref | ref |
| Yes | 21 |  |  | 0.4 (0.2, 0.6) | 51% (23%, 84%) |
| Predominant source of milk during infancy |  |  |  |  |  |
| Infant Formula^a^ | 11 |  |  | ref | ref |
| Breastmilk^b^ | 11 |  |  | -0.4 (-0.6, -0.2) | -34% (-46%, -20%) |
| Mix of Breastmilk and Formula^c^ | 6 |  |  | -0.2 (-0.4, 0.1) | -17% (-34%, 4%) |
| ^*^Prenatal correlate of breastfeeding and formula variables not analyzed since correlates occur after tooth Sr was measured  † Applies to index (participating) child only  ^a^ Predominantly formula fed defined as maternal report of being breastfed less than 6 months & consuming > 50% formula during first year of life  ^b^ Predominantly breastfed defined as maternal report of being breastfed longer than 6 months & consuming less than 50% formula during first year of life  ^c^ Mix of breastmilk and formula defined as maternal report of being breastfed for less than 6 months & consuming less than 50% formula during first year of life OR maternal report of being breastfed for longer than 6 months & consuming > 50% formula during first year of life | | | | | |

| **Table S13**: Correlates of log-transformed prenatal and postnatal dentine Zn levels (as ^66^Zn^43^Ca) from sex-adjusted linear mixed models | | | | | |
| --- | --- | --- | --- | --- | --- |
|  |  | Prenatal Zn | | Postnatal Zn | |
|  | N | β (95% CI) | % Difference  (95% CI) | β (95% CI) | % Difference  (95% CI) |
| Child sex |  |  |  |  |  |
| Male | 18 | ref | ref | ref | ref |
| Female | 10 | -0.01 (-0.1, 0.1) | -1% (-9%, 8%) | -0.1 (-0.2, 0.1) | -5% (-16%, 8%) |
| Child age, continuous (years) | - | 0.01 (-0.01, 0.03) | 1% (-1%, 3%) | 0.02 (-0.01, 0.04) | 2% (-1%, 4%) |
| Birth order |  |  |  |  |  |
| Second or higher | 15 | ref | ref | ref | ref |
| First | 13 | 0.02 (-0.1, 0.1) | 2% (-7%, 11%) | 0.03 (-0.1, 0.2) | 3% (-10%, 17%) |
| Well type |  |  |  |  |  |
| Private | 3 | ref | ref | ref | ref |
| Public | 25 | -0.02 (-0.2, 0.1) | -2% (-14%, 11%) | -0.05 (-0.3, 0.2) | -5% (-22%, 16%) |
| Tooth type |  |  |  |  |  |
| Incisor | 24 | ref | ref | ref | ref |
| Molar | 4 | -0.02 (-0.1, 0.1) | -2% (-13%, 10%) | 0.04 (-0.1, 0.2) | 4% (-12%, 24%) |
| Anemic while pregnant |  |  |  |  |  |
| No | 19 | ref | ref | ref | ref |
| Yes | 8 | -0.02 (-0.2, 0.1) | -3% (-18%, 13%) | 0.1 (-0.2, 0.4) | 10% (-14%, 40%) |
| Use of filter during pregnancy |  |  |  |  |  |
| Yes | 14 | ref | ref | ref | ref |
| No | 14 | 0.04 (-0.05, 0.1) | 4% (-4%, 12%) | -0.002 (-0.1, 0.1) | -0.3% (-12%, 13%) |
| Filtered water use for food prep |  |  |  |  |  |
| Always | 3 | ref | ref | ref | ref |
| Rarely / Sometimes | 4 | -0.04 (-0.2, 0.1) | -4% (-20%, 14%) | -0.1 (-0.4, 0.2) | -9% (-30%, 17%) |
| Never | 21 | 0.01 (-0.1, 0.1) | 1% (-12%, 15%) | 0.1 (-0.2, 0.3) | 5% (-14%, 28%) |
| Filtered water use for coffee/tea prep |  |  |  |  |  |
| Always | 6 | ref | ref | ref | ref |
| Rarely / Sometimes | 6 | 0.1 (-0.1, 0.2) | 5% (-8%, 20%) | 0.1 (-0.2, 0.3) | 6% (-14%, 29%) |
| Never | 16 | 0.02 (-0.1, 0.1) | 2% (-9%, 13%) | 0.04 (-0.1, 0.2) | 4% (-11%, 21%) |
| Bottled water use during pregnancy |  |  |  |  |  |
| Always | 2 | ref | ref | ref | ref |
| Rarely / Sometimes | 17 | 0.003 (-0.2, 0.2) | 0% (-15%, 19%) | -0.01 (-0.3, 0.3) | -1% (-23%, 29%) |
| Never | 9 | 0.02 (-0.2, 0.2) | 2% (-19%, 18%) | -0.03 (-0.3, 0.3) | -3% (-28%, 29%) |
| Ever breastfed*† |  |  |  |  |  |
| No | 3 |  |  | ref | ref |
| Yes | 25 |  |  | -0.2 (-0.4, -0.03) | -20% (-34%, -4%) |
| Ever use formula*† |  |  |  |  |  |
| No | 7 |  |  | ref | ref |
| Yes | 21 |  |  | 0.04 (-0.1, 0.2) | 4% (-10%, 21%) |
| Predominant source of milk during infancy |  |  |  |  |  |
| Infant Formula^a^ | 11 |  |  | ref | ref |
| Breastmilk^b^ | 11 |  |  | -0.1 (-0.2, 0.1) | -5% (-17%, 10%) |
| Mix of Breastmilk and Formula^c^ | 6 |  |  | -0.03 (-0.2, 0.2) | -3% (-18%, 15%) |
| ^*^Prenatal correlate of breastfeeding and formula variables not analyzed since correlates occur after tooth Zn was measured  † Applies to index (participating) child only  ^a^ Predominantly formula fed defined as maternal report of being breastfed less than 6 months & consuming > 50% formula during first year of life  ^b^ Predominantly breastfed defined as maternal report of being breastfed longer than 6 months & consuming less than 50% formula during first year of life  ^c^ Mix of breastmilk and formula defined as maternal report of being breastfed for less than 6 months & consuming less than 50% formula during first year of life OR maternal report of being breastfed for longer than 6 months & consuming > 50% formula during first year of life | | | | | |

| **Table 14:**  Correlates of log-transformed postnatal dentine, truncated to 20 weeks, Ba, Mn and Pb levels from sex-adjusted linear mixed models | | | | | | | |
| --- | --- | --- | --- | --- | --- | --- | --- |
|  |  | Postnatal Ba (up to 20 weeks) | | Postnatal Mn (up to 20 weeks) | | Postnatal Pb (up to 20 weeks) | |
|  | N | β (95% CI) | % Difference (95% CI) | β (95% CI) | % Difference (95% CI) | β (95% CI) | % Difference (95% CI) |
| Child sex |  |  |  |  |  |  |  |
| Male | 18 | ref | ref | ref | ref | ref | ref |
| Female | 10 | 0.1 (-0.3, 0.5) | 11% (-22%, 58%) | 0.9 (0.2, 1.6) | 141% (25%, 366%) | 0.4 (-0.2, 1.0) | 54% (-13%, 171%) |
| Age (continuous), years |  | 0.01 (-0.1, 0.1) | 1% (-6%, 9%) | -0.1 (-0.3, 0.1) | -10% (-23%, 5%) | -0.1 (-0.2, 0.1) | -5% (-16%, 8%) |
| Birth order |  |  |  |  |  |  |  |
| Second or higher | 15 | ref | ref | ref | ref | ref | ref |
| First | 13 | -0.01 (-0.4, 0.4) | -1% (-32%, 45%) | 0.2 (-0.5, 0.9) | 23% (-39%, 149%) | -0.1 (-0.7, 0.5) | -9% (-50%, 66%) |
| Tooth type |  |  |  |  |  |  |  |
| Incisor | 24 | ref | ref | ref | ref | ref | ref |
| Molar | 4 | 0.02 (-0.5, 0.5) | 2% (-37%, 66%) | 0.2 (-0.8, 1.3) | 27% (-53%, 247%) | 0.2 (-0.6, 1.1) | 24% (-44%, 175%) |
| Well type |  |  |  |  |  |  |  |
| Private | 3 | ref | ref | ref | ref | ref | ref |
| Public | 25 | -0.3 (-0.9, 0.3) | -28% (58%, 25%) | 0.2 (-0.9, 1.3) | 20% (-58%, 241%) | -0.1 (-1.0, 0.9) | -5% (-61%, 131%) |
| Anemic while pregnant |  |  |  |  |  |  |  |
| No | 19 | ref | ref | ref | ref | ref | ref |
| Yes | 8 | 0.1 (-0.4, 0.5) | 6% (-28%, 56%) | -0.2 (-1.0, 0.6) | -19% (-62%, 70%) | 0.5 (-0.2, 1.1) | 62% (-11%, 196%) |
| Use of filter during pregnancy |  |  |  |  |  |  |  |
| Yes | 14 | ref | ref | ref | ref | ref | ref |
| No | 14 | 0.2 (-0.2, 0.5) | 22% (-13%, 70%) | 0.3 (-0.4, 1.1) | 39% (-31%, 180%) | 0.1 (-0.5, 0.7) | 10% (-38%, 93%) |
| Filtered water use for food prep during pregnancy |  |  |  |  |  |  |  |
| Always | 3 | ref | ref | ref | ref | ref | ref |
| Rarely / Sometimes | 4 | -0.2 (0.6, 1.0) | 24% (-42%, 163%) | 0.3 (-1.1, 1.8) | 40% (-65%, 450%) | -1.1 (-2.3, 0.03) | -67% (-89%, -3%) |
| Never | 21 | 0.1 (-0.5, 0.8) | 16% (-35%, 109%) | -0.3 (-1.4, 0.8) | -24% (-74%, 119%) | -1.0 (-1.9, -0.1) | -65% (-85%, 17%) |
| Filtered water use for coffee/tea prep during pregnancy |  |  |  |  |  |  |  |
| Always | 6 | ref | ref | ref | ref | ref | ref |
| Rarely / Sometimes | 6 | 0.5 (-0.1, 1.0) | 64% (-2%, 177%) | 0.3 (-0.8, 1.4) | 33% (-54%, 282%) | -0.2 (-1.2, 0.7) | -20% (-67%, 97%) |
| Never | 16 | -0.01 (-0.4, 0.4) | -1% (-34%, 48%) | -0.2 (-1.0, 0.7) | -14% (-62%, 95%) | -0.4 (-1.1, 0.4) | -31% (-66%, 38%) |
| Bottled water use during pregnancy |  |  |  |  |  |  |  |
| Always | 2 | ref | ref | ref | ref | ref | Ref |
| Rarely / Sometimes | 17 | 0.2 (-0.6, 0.9) | 19% (-43%, 146%) | -0.1 (-1.6, 1.3) | -11% (-78%, 265%) | 0.3 (-0.9, 1.5) | 37% (-57%, 342%) |
| Never | 9 | 0 (-0.8, 0.9) | 0% (-55%, 125%) | -0.4 (-2.0, 1.2) | -34% (-86%, 200%) | 0.1 (-1.2, 1.5) | 14% (-69%, 314%) |
| Ever breastfed† |  |  |  |  |  |  |  |
| No | 3 | ref | ref | ref | ref | ref | ref |
| Yes | 25 | -0.7 (-1.2, -0.1)* | -48% (-69%, -12%)* | 0.7 (-0.5, 1.8) | 92% (-33%, 449%) | 0.1 (-0.9, 1.1) | 12% (-56%, 181%) |
| Ever use formula† |  |  |  |  |  |  |  |
| No | 7 | ref | ref | ref | ref | ref | ref |
| Yes | 21 | 0.6 (0.2, 1.0)* | 81% (28%, 155%)* | -0.1 (-0.9, 0.7) | -8% (-57%, 100%) | 0 (-0.7, 0.6) | -5% (-51%, 85%) |
| Predominant source of milk during infancy |  |  |  |  |  |  |  |
| Infant Formula^a^ | 11 | ref | ref | ref | ref | ref | ref |
| Breastmilk^b^ | 11 | -0.5 (-0.9, 0.1)* | -40% (-58%, -14%)* | 0.3 (-0.5, 1.1) | 30% (-39%, 178%) | 0.1 (-0.6, 0.7) | 8% (-42%, 101%) |
| Mix of Breastmilk and Formula^c^ | 6 | -0.2 (-0.6, 0.3) | -15% (-44%, 30%) | 0.3 (-0.7, 1.2) | 34% (-46%, 230%) | 0.6 (-0.2, 1.4) | 80% (-14%, 277%) |
| † Applies to index (participating) child only  ^a^ Predominantly formula fed defined as maternal report of being breastfed less than 6 months & consuming > 50% formula during first year of life  ^b^ Predominantly breastfed defined as maternal report of being breastfed longer than 6 months & consuming less than 50% formula during first year of life  ^c^ Mix of breastmilk and formula defined as maternal report of being breastfed for less than 6 months & consuming less than 50% formula during first year of life OR maternal report of being breastfed for longer than 6 months & consuming > 50% formula during first year of life | | | | | | | |
